# Characterization of a live-attenuated HCMV-based vaccine platform

**DOI:** 10.1038/s41598-019-55508-w

**Published:** 2019-12-17

**Authors:** Patrizia Caposio, Sjoerd van den Worm, Lindsey Crawford, Wilma Perez, Craig Kreklywich, Roxanne M. Gilbride, Colette M. Hughes, Abigail B. Ventura, Robert Ratts, Emily E. Marshall, Daniel Malouli, Michael K. Axthelm, Daniel Streblow, Jay A. Nelson, Louis J. Picker, Scott G. Hansen, Klaus Früh

**Affiliations:** 1 Vaccine and Gene Therapy Institute, Oregon Health & Science University, Beaverton, Oregon 97006 USA; 2 Present Address: Batavia Biosciences B.V., Zernikedreef 16, 2333 CL Leiden, Netherlands; 3 Present Address: Vir Biotechnology, 4640, SW Macadam Avenue, Portland, OR 97239 USA

**Keywords:** Live attenuated vaccines, Herpes virus

## Abstract

Vaccines based on cytomegalovirus (CMV) demonstrate protection in animal models of infectious disease and cancer. Vaccine efficacy is associated with the ability of CMV to elicit and indefinitely maintain high frequencies of circulating effector memory T cells (T_EM_) providing continuous, life-long anti-pathogen immune activity. To allow for the clinical testing of human CMV (HCMV)-based vaccines we constructed and characterized as a vector backbone the recombinant molecular clone TR3 representing a wildtype genome. We demonstrate that TR3 can be stably propagated *in vitro* and that, despite species incompatibility, recombinant TR3 vectors elicit high frequencies of T_EM_ to inserted antigens in rhesus macaques (RM). Live-attenuated versions of TR3 were generated by deleting viral genes required to counteract intrinsic and innate immune responses. In addition, we eliminated subunits of a viral pentameric glycoprotein complex thus limiting cell tropism. We show in a humanized mouse model that such modified vectors were able to establish persistent infection but lost their ability to reactivate from latency. Nevertheless, attenuated TR3 vectors preserved the ability to elicit and maintain T_EM_ to inserted antigens in RM. We further demonstrate that attenuated TR3 can be grown in approved cell lines upon elimination of an anti-viral host factor using small interfering RNA, thus obviating the need for a complementing cell line. In sum, we have established a versatile platform for the clinical development of live attenuated HCMV-vectored vaccines and immunotherapies.

## Introduction

Vaccines using cytomegalovirus (CMV) as antigen-carrying vectors have shown great promise in animal models of infectious diseases and cancer^1–3^. Using rhesus CMV (RhCMV) as our model system we demonstrated unprecedented control of infection by highly pathogenic SIV upon repeated low dose challenge^4,5^. Moreover, we recently also reported that RhCMV-vectors expressing antigens derived from *Mycobacterium tuberculosis* (TB) protected against intrabronchial challenge with TB to which RM are exquisitely susceptible^6^. Finally, we demonstrated that RhCMV-based vaccines eliciting T cells against antigens of the malaria parasite *Plasmodium knowlesi* strongly reduced the release of liver stage parasites into the blood^7^. Taken together these studies demonstrate that CMV-vectors represent a novel vaccine platform for many applications.

Since RhCMV-based vectors elicit little to no antibody responses to the inserted antigens, the protection elicited by these vectors is almost certainly attributable to cellular immunity^4,6,7^. Indeed, one of the most unique aspects of RhCMV-based vectors is their ability to elicit and indefinitely maintain high frequencies of circulating and tissue-resident effector memory CD4+ and CD8+ T cells (T_EM_) to the inserted antigens^4,5^. The likely mechanism of T cell mediated protection was illustrated in the SIV model where 50% of RhCMV/SIV vaccinated animals were initially infected with SIV, as documented by cell-associated, replication-competent SIV and/or by the development of T cell responses to SIV antigens not included in the vaccine. However, animals remained aviremic and went on to eventually clear the SIV infection to below detection limits of all available virological measurements^5^. A very similar result was obtained when anti-retroviral treatment was started within 4–5 days of SIV challenge strongly suggesting that RhCMV/SIV elicited T cell immunity provided an early intercept of SIV infection that prevents the seeding of a long-lived latent SIV reservoir^8^. Thus, CMV-elicited T_EM_ provide a rapid interception and control of pathogens at the portal of pathogen entry and maintain control over time.

Since T cell effector differentiation is antigen-driven, it is likely that CMV-induced T_EM_ are maintained by continuous or recurring antigen exposure due to viral persistence and reactivation in antigen presenting cells (APC)^9^. Surprisingly however, this immune stimulation does not seem to require viral dissemination within the host as long as latency is established. In murine models it was shown previously that MCMV deleted for essential viral genes was still able to elicit and maintain T_EM_ despite being spread-deficient^10,11^. More recently, we demonstrated that RhCMV lacking the tegument protein pp71 is highly debilitated in its ability to spread and was no longer transmitted either through secretions or by blood transfusions^12^. Nevertheless, above a given dose threshold, pp71-deleted RhCMV elicited immune responses that retained all features described above^12^. Moreover, pp71-deleted RhCMV/SIV vaccines protected against homologous and heterologous challenge with SIV and most of the protected animals were able to control SIV infection again when re-challenged years later^13^.

CMV species co-evolved with their individual host species and no naturally occurring instances of cross species infections have been observed^14^. Thus, CMV vectors have to be based on a HCMV vector backbone to maintain the desired immunological features of CMV-based vectors for human vaccines and immunotherapies. Since disseminating HCMV can cause serious disease in individuals with an immature or compromised immune system^15^, HCMV-based vaccine vectors intended for general prophylactic use in human need to be attenuated. Spread-deficient animal CMV species that maintain all unique T cell immunity features thus provide a blueprint for the design of highly attenuated HCMV-vectors for human use. To permit the genetic modifications required to insert heterologous antigens as well as safety features the selected HCMV strain needs to be amenable for genetic manipulation while maintaining genetic stability and manufacturability. Here we describe the novel HCMV-based vaccine platform TR3 that, starting from a complete viral genome representative of the low passage isolate HCMV TR^16^, can be genetically modified to introduce heterologous antigens as well as specific deletions that impact vector safety and immunogenicity. Using a humanized mouse model system we demonstrate that deletion or inactivation of the pp71-encoding gene UL82 renders TR3 reactivation-deficient while maintaining the ability to establish latency. We further demonstrate that TR3 carrying heterologous antigens can elicit and maintain T_EM_ to these antigens in RM even when deleted for pp71. We further show that pentameric complex (PC)-deleted TR3 maintains vector function *in vivo* either alone or in combination with pp71 deletion. Thus, we developed a robust and versatile live attenuated vaccine vector platform that is suitable for clinical testing.

## Results

### Construction and characterization of the HCMV-TR3 vector backbone

**Figure 1:**
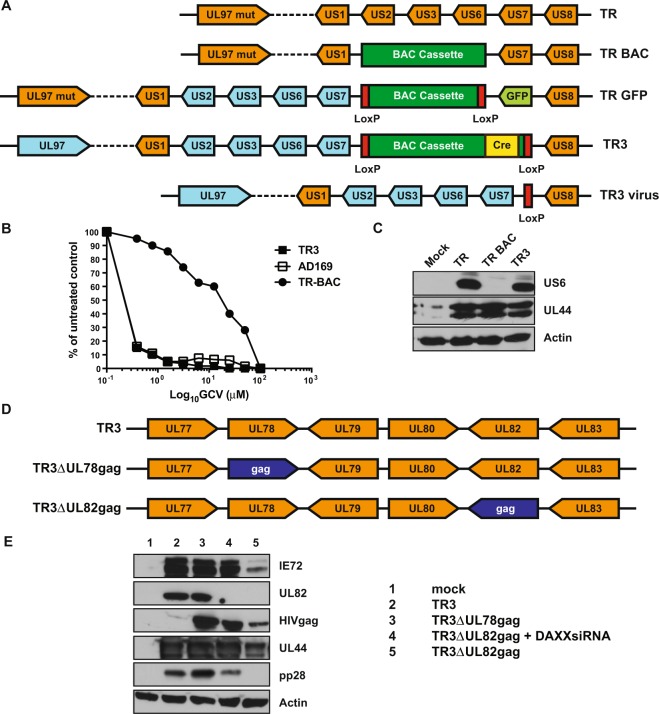
Construction and *in vitro* characterization of HCMV TR3. (**A**) Sequential construction steps resulting in TR3. The original isolate TR^72^ was cloned by replacing the US2-6 region with a BAC cassette resulting in TR-BAC^16^. TR-GFP was generated by inserting loxP sites flanking the BAC cassette, replacing US7 with a GFP expression cassette and inserting the US2-7 region of AD169^18^. TR3 was generated from TR-GFP by deleting the GFP cassette, inserting a Cre-expression cassette into the BAC cassette and by replacing the defective UL97 with intact UL97 of AD169. The BAC cassette in TR3 is self-excising resulting in a residual loxP site upon reconstitution of virus. (**B**) TR3 is sensitive to Ganciclovir. Human MRC-5 fibroblasts were infected with the indicated viruses at MOI = 0.5 in the presence of decreasing concentrations of Ganciclovir. Culture supernatants were harvested when controls showed full cytopathic effect and the amount of viral progeny produced was quantified by standard plaque assay. Results are shown as percent of untreated control. (**C**) US6 expression by TR3. MRC-5 cells were infected or mock infected with the indicated viruses at MOI = 0.5. At 96 h after infection, cells were harvested and cell lysates were electrophoretically separated and probed for expression of the indicated viral or host proteins by immunoblot. The blots for each protein are shown as cropped from different parts of the same gel. (**D**) Design of TR3ΔUL78gag and TR3ΔUL82gag. (**E**) Viral protein expression in the absence of pp71. MRC-5 cells were infected or mock infected with the indicated viruses at MOI = 0.5 in the presence or absence of DAXX-targeting siRNA. Cells were harvested at 96 h post-infection. Cell lysates were electrophoretically separated and probed for the indicated viral and cellular proteins by immunoblot. The blots for each protein are shown as cropped from different parts of the same or different gels separating the same lysate. HIVgag was detected using a polyclonal antiserum to the p24 subunit.

To translate the RhCMV-vector results into clinical testing we selected an HCMV strain that was likely to establish persistent infection since we posit that viral persistence is required to elicit and maintain potent T_EM_-responses. At the same time the vector needed to be amenable to the genetic manipulations essential for the insertion of heterologous antigens as well as the introduction of safety and immunogenicity features. Low passage HCMV strains have the highest likelihood of maintaining the ability of the parental isolate to persist upon infection. However, many clinical isolates are genetically unstable upon prolonged passage in tissue culture rendering them unsuitable as a vaccine platform^17^. We previously described the clinical isolate HCMV TR as being representative for low passage isolates while lacking typical genetic adaptations to tissue culture^16^. To evaluate the suitability of HCMV TR as a vaccine vector backbone we modified a previously described recombinant that was cloned as a bacterial artificial chromosome (BAC) which facilitates genetic manipulation^16^. In this original clone (TR-BAC) a non-excisable BAC-cassette containing the bacterial origin of replication and a bacterial resistance marker was inserted into the US2-6 genomic region thus deleting parts of US2 and US6, and all of US3, US4 and US5^16^ (Fig. 1A). To restore expression of the US2-6 genes, Lauron *et al*. inserted the US2-US7 region from HCMV strain AD169 as well as loxP sites to allow the excision of the BAC cassette upon Cre-expression in mammalian cells. In addition, a GFP-expression cassette was inserted downstream of US8, thereby deleting the TR US7 gene^18^ (Fig. 1A). Since the US2-11 homologous genomic region in RhCMV was shown to be important for superinfection^19^ we used this US2-7-repaired version (TR-GFP) for further modifications. Using the galK BAC recombineering system^20^ we deleted the GFP marker and inserted the *Cre* recombinase gene into the BAC cassette which renders the cassette self-excising upon viral reconstitution^21^. Since HCMV TR was resistant to Ganciclovir (GCV) due to mutations in the viral kinase UL97 that is required to phosphorylate the pro-drug we replaced the gene with UL97 from AD169 which is GCV sensitive (Fig. 1A). The final BAC, termed TR3, was sequenced by next generation sequencing (NGS) (Fig. S1). Upon reconstitution in human fibroblasts, viral genomes were also sequenced by NGS which confirmed the excision of the BAC cassette as well as the presence of US2-7 region of AD169, a loxP site between US7 and US8, and the UL97 gene from AD169 (Fig. S2). To demonstrate that insertion of AD169 UL97 conferred GCV sensitivity to TR3, we performed a plaque reduction assay in fibroblasts in the presence of increasing GCV concentrations (Fig. 1B). We next verified expression of US6, an inhibitor of the peptide transporter associated with antigen presentation^22^, that is encoded in the AD169-derived US2-7 region (Fig. 1C). Expression levels were comparable with the US6 expression observed in the original clinical isolate TR whereas US6 is absent from the original BAC-cloned TR-BAC. Taken together these data demonstrate that we successfully reconstructed a vector backbone with a genome configuration that is representative of low passage HCMV clinical isolates. A single loxP site is the only remaining heterologous sequence upon viral reconstitution *in vitro* (Figs. S1, S2).

### Generation of a live-attenuated HCMV vector platform by deletion or inactivation of UL82 (pp71)

Given the possibility of HCMV to cause disease in individuals with an immature or compromised immune system, HCMV-based vectors need to be attenuated in order to be safe for use as a vaccine platform. We recently reported that RhCMV lacking the viral tegument protein pp71 was highly attenuated *in vivo*, as evident from reduced dissemination, lack of shedding as well as lack of natural transmission or transmission upon transfusion^12^. Nevertheless, above the effective dose threshold, pp71-deleted RhCMV elicited and maintained T_EM_ responses that were similar to that of the parental strain and these immune responses were able to confer long-term protection against homologous and heterologous challenge with highly pathogenic SIV_mac239_^13^. Based on these results, we selected deletion of pp71 as our primary attenuation strategy. While pp71-deleted RhCMV can be recovered upon transfection of BAC into fibroblasts without the need of complementation^12^, it was previously reported that pp71-deleted HCMV could only be recovered in fibroblasts expressing exogenous pp71^23^. A major function of pp71 is to counteract the host repressor DAXX (Death-domain associated protein 6) which otherwise limits viral immediate early (IE) gene expression^24–26^. Since IE expression can be restored in cells infected with pp71-deleted HCMV treated with small interfering RNA (siRNA) targeting DAXX^25^ we hypothesized that it is possible to recover and grow UL82-deleted TR3 in the presence of siRNA targeting DAXX. This alternative strategy would obviate the need for a complementing cell line. To generate a UL82-deleted HCMV we replaced the pp71-encoding ORF with that of HIVgag (Fig. 1D) a strategy we have used in the past to express SIV antigens in RhCMV^12,13^. In the pp71-intact control vector we replaced UL78, a viral G protein coupled receptor not required for growth in fibroblasts^27^, with HIVgag thus using the UL78 promoter to drive HIVgag expression (Fig. 1D). We previously reported the induction of SIV-antigen specific T cell responses by RhCMV expressing an SIV antigen in the UL78-homologous gene of RhCMV^12^.

The sequences of the BACs for TR3ΔUL78gag and TR3ΔUL82gag were confirmed by NGS (Fig. S1). We were able to recover virus when siRNA targeting DAXX was transfected into fibroblasts together with the BAC for TR3ΔUL82gag (Fig. 1E). To monitor the impact of pp71 deletion on viral protein expression we monitored viral protein expression as well as the expression of HIVgag by immunoblotting of cell lysates obtained 6 days post infection (dpi) with each individual recombinant virus. In cells infected with TR3ΔUL82gag in the absence of DAXX siRNA we did not detect pp71 expression or the late protein pp28 whereas low amounts of IE72 and the E protein UL44 were observed (Fig. 1E). In contrast, treatment with DAXX siRNA resulted in protein expression levels comparable to pp71-intact TR3. Importantly, HIVgag expression was observed in the absence of pp71 and even when expression was driven by the UL82 promoter although levels were lower compared to pp71-expressing controls. These data indicate that HIVgag might continue to be expressed at a low level *in vivo* even when viral replication is severely attenuated in the absence of DAXX siRNA.

### ***In vitro*** growth of live-attenuated TR3

**Figure 2:**
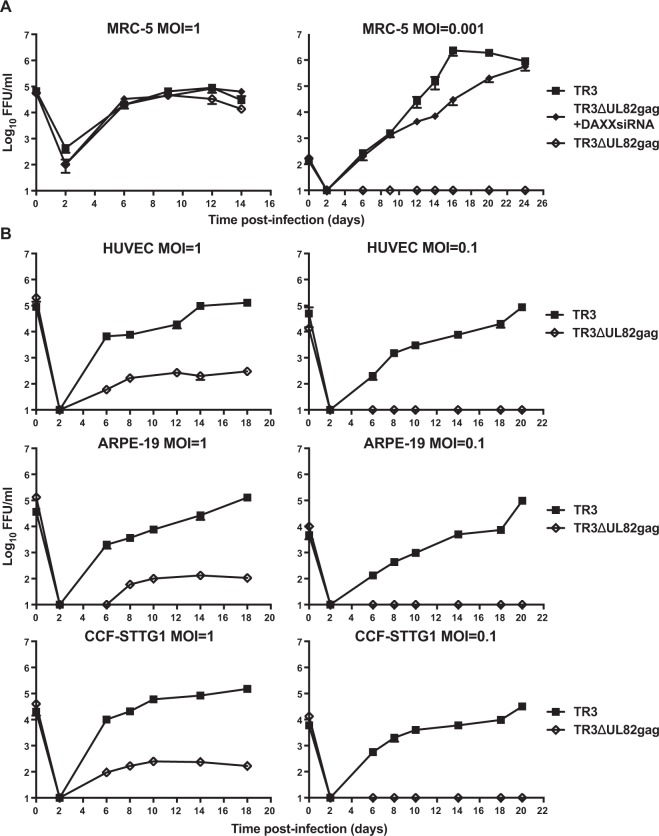
*In vitro* growth of pp71-deficient HCMV TR3. (**A**) MOI-dependent growth deficiency of UL82-deleted TR3 in fibroblasts. MRC-5 cells were inoculated with TR3 or TR3ΔUL82gag at a multiplicity of infection (MOI) of 1 or 0.001 and cell culture supernatants were collected at the indicated times. Where indicated cells were transfected with 10 nM of Daxx siRNA and, after 24 h, infected with HCMV. After 2 h, the inoculum was removed, cells were washed 3 times with PBS and fresh media was added. A second round of transfection was performed at day 10 post-infection. Virus was titered on BJ-pp71 cells. The average titer of triplicate experiments (+/− SD) is shown. **(B**) Growth deficiency of UL82-deleted TR3 in non-fibroblasts. Endothelial cells (HUVEC), epithelial cells (ARPE-19) and astrocytic cells (CCF-STTG1) were infected with the indicated MOI of TR3 or TR3ΔUL82gag. The supernatants were harvested at the indicated days and the virus titer determined on BJ-pp71 cells.

To further characterize the growth defect of pp71-deficient TR3 in different cell lines we needed a cell line that allows for the accurate titration of UL82-deleted HCMV. To this end, we introduced a UL82 expression vector into human foreskin BJ fibroblasts that are life-extended by telomerase expression^28^ (see Methods). To accurately determine the titers of pp71-deficient virus stocks or culture supernatants we monitored the expression of viral IE proteins in BJ-pp71 cells by immunocytochemistry. Using this assay we thus compared *in vitro* growth of pp71-intact and pp71-deficient TR3 at different MOIs upon infection of MRC-5 cells. As shown in Fig. 2A transfection of the MRC-5 cells with DAXX siRNA resulted in final titers, determined as focus forming units (FFU), of TR3ΔUL82gag that were comparable to pp71-intact TR3ΔUL78gag even at a very low MOI = 0.001. As reported previously for AD169, deletion of pp71 from TR3 did not impact viral growth at high MOI^23^. The impact of pp71 deletion on TR3 growth was further investigated in endothelial, epithelial, and astrocytic cells. HUVEC, ARPE-19, and CCF-STTG1 cells were infected at high and low MOI with TR3 or TR3ΔUL82gag in the absence of DAXX siRNA. In all three cell types we observed a growth defect even at high MOI = 1 and no viral growth at MOI = 0.1 (Fig. 2B). Taken together these data demonstrate that the absence of pp71 profoundly affects growth of TR3 *in vitro* consistent with previously reported growth defects of AD169.

### Stability of live-attenuated HCMV TR3 upon repeated passaging *in vitro*

Most clones of low passage HCMV isolates harbouring complete viral genomes rapidly mutate *in vitro*, including point mutations, gene deletions and rearrangements of sub-genomic regions^29^. Such tissue culture adaptations have rendered it difficult if not impossible to use complete viral genomes as backbones for vectors or vaccines. This is particularly true when growing clinical isolates in human fibroblasts where it was shown that upon transfection of a full-length BAC clone of the clinical isolate Merlin the viral progeny was rapidly selected for mutations in the gene RL13 followed by loss of the UL128, UL130 or UL131 subunits of the pentameric complex (PC)^17^. Similarly, we observed that the PC was rapidly lost upon reconstitution of a BAC-cloned version of the clinical isolate Toledo in fibroblasts^30^. The PC, consisting of the viral proteins gH/gL/UL128/UL130/UL131, facilitates entry into non-fibroblast cells such as endothelial cells, epithelial cells and myeloid cells by endocytosis^31,32^. Host cell proteins that are specifically expressed in these cells, including neuropilin-2, ORF14I1 and CD147, have been identified in playing a crucial role in PC-mediated entry^33–35^.

**Figure 3:**
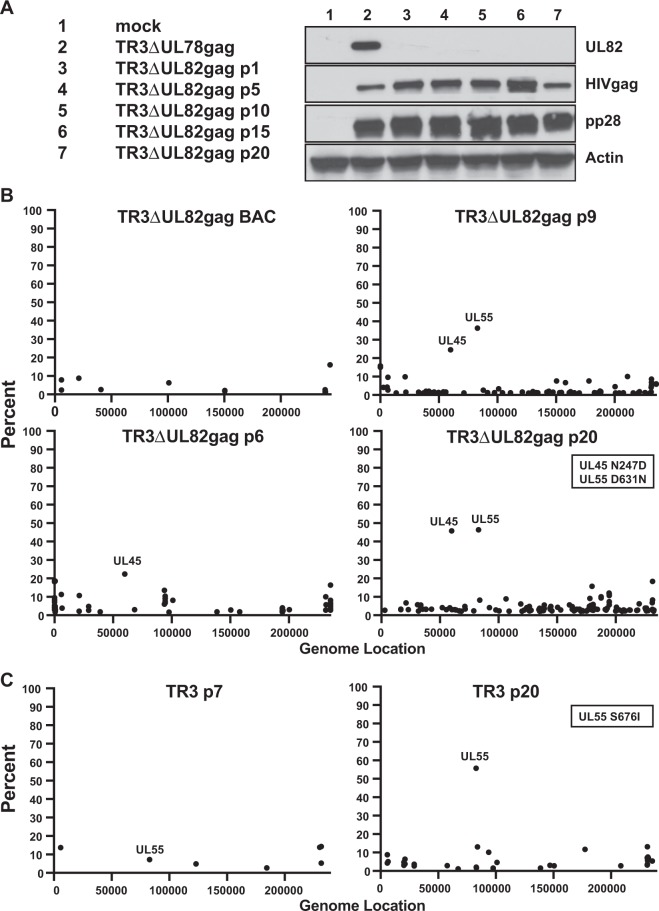
Genetic stability of pp71 deficient HCMV TR3. (**A**) Stable expression of HIVgag upon multiple passages. MRC-5 cells were mock-infected or infected with TR3ΔUL78gag or TR3ΔUL82gag at an MOI of 0.01 and the supernatant was harvested at full CPE. For sequential passages (p1-p20), TR3ΔUL82gag in the supernatant was titered on BJ-pp71 cells and used for the next round of infection at MOI = 0.01. Lysates from cells harvested at the indicated passages were electrophoretically separated and probed for expression of the shown viral proteins or cellular actin by immunoblot. The blots for each protein are shown as cropped from different parts of the same gel. (**B**) SNP analysis of TR3ΔUL82gag upon serial passaging. Viral DNA was harvested at the indicated passages from the same samples as in (**A**) and subjected to Next Generation Sequencing (NGS) on a MiSeq platform. The position and frequency of SNPs are shown along the viral genome. Two SNPs were enriched upon passaging: T-C at position 59972 and C-T at position 82959 resulting in an Asn-Asp change in UL45 and an Asp-Asn change in UL55. (**C**) SNP analysis of TR3 upon serial passaging. TR3 was serially passaged as in (**A**) and viral DNA was harvested at the indicated passages and subjected to NGS. The position and frequency of SNPs are shown along the viral genome. One SNP was enriched upon passaging: C-A at position 82489 resulting in a Ser-Ile change in UL55.

To evaluate the genetic integrity of our live attenuated HCMV platform we monitored the viral genomic sequences of TR3ΔUL82gag over a series of 20 passages in MRC-5 fibroblasts. MRC-5 cells were infected at each passage with TR3ΔUL82gag in the presence of DAXX siRNA at an MOI = 0.01. After every 5 passages cells and supernatants were harvested and analyzed by immunoblot whereas passages 6, 9 and 20 were analyzed by NGS. Immunoblot analysis revealed stable expression of HIVgag over all passages (Fig. 3A**)**. Genomic sequence analysis indicated that each passage increased the number of single nucleotide polymorphisms (SNPs) which is expected from a replicating viral genome resulting in random, non-debilitating mutations. However, most of these SNPs remained at low frequency with the exception of two SNPs in glycoprotein B (gB, UL55) and in nucleotide reductase (UL45) which increased in frequency in later passages (Fig. 3B). Similarly, we observed an enrichment of a different SNP in gB when unmodified TR3 was passaged 20 times (Fig. 3C) The impact of these SNPs on gB function has not been evaluated, but since gB is an essential gene these mutations are unlikely to be detrimental for viral growth. Interestingly, it was recently reported that the gB of the highly passaged AD169 strain contains a SNP (D275Y) that seems to accelerate viral entry into fibroblasts by membrane fusion^36^. Conceivably, the SNPs observed in TR3 gB might thus reflect a similar adaptation to fibroblast growth. However, TR3 clearly did not seem to undergo the same selection pattern described for other clinical isolates that result in the rapid selection of RL13 and PC mutations upon passaging in fibroblasts. Moreover, we did not observe any deletions or any major genomic rearrangements despite extensive passaging. Based on these results we conclude that TR3 is a genetically stable vector platform that retains the genomic configuration of a low passage clinical isolate.

### Pp71-deficient HCMV-TR3 establishes a persistent infection but does not reactivate in humanized mice

**Figure 4:**
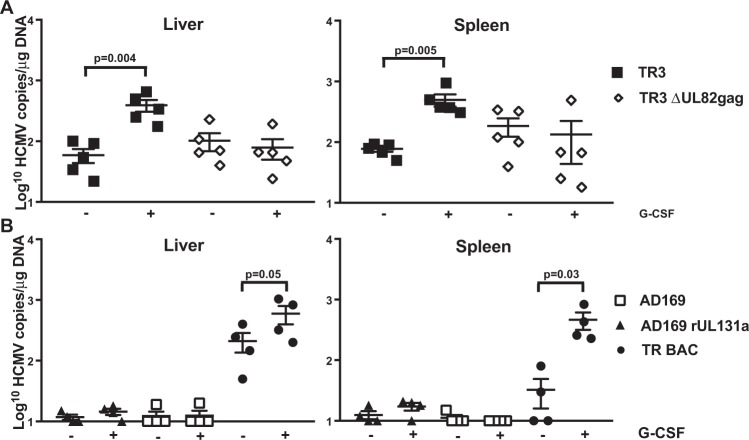
pp71-deficient TR3 establishes latency, but does not reactivate *in vivo*. (**A**) Latency and reactivation of TR3 and pp71-deleted TR3 in humanized mice. NOD-*scid*IL2Rγc null (NSG) mice engrafted with human CD34+ stem cells (n = 10 per group) were inoculated IP with MRC-5 fibroblasts infected with TR3 or TR3ΔUL82gag. Eight weeks post‐infection, human hematopoietic stem cells were mobilized by G-CSF treatment and the viral load was measured in liver and spleen one week later. The viral DNA copy number was determined by quantitative PCR and is shown per microgram of total DNA. Values in the absence of granulocyte colony stimulating factor (G-CSF) represent the latent viral load and values after G-CSF stimulation represent the reactivation of virus emerging from latency. (**B**) Latency and reactivation of PC-deficient and PC-intact AD169 compared to TR-BAC in humanized mice. NSG mice were inoculated with fibroblasts infected with AD169 and AD169rUL131a^71^, or TR-BAC^16^ and genome copy numbers were determined as in (**A**). Statistical significance was determined using two-way analysis of variance, followed by Bonferroni’s posthoc test (P values are shown).

We previously demonstrated that HCMV TR and other clinical isolates can establish persistent infection in a humanized mouse model in which human CD34+ hematopoietic precursor cells (HPC) are engrafted into NOD-*scid*IL2Rγc null (NSG) mice^37–40^. Moreover, mobilization of human monocyte/macrophages by granulocyte-colony stimulating factor (G-CSF) reactivates virus in peripheral organs^39^. This model thus represented an opportunity to evaluate the attenuation of pp71-deficient TR3 *in vivo*. HuNSG mice (n = 10/group) were inoculated intraperitoneally with human fibroblasts infected with TR3, or TR3ΔUL82gag and, after 8 weeks, 5 animals in each group were treated with G-CSF. One week later, animals were sacrificed and viral genome copy numbers were determined in spleen and liver by quantitative PCR. As reported previously for the parental TR strain^39^, latent TR3 was detected in spleen and liver and viral genome copy numbers increased upon G-CSF treatment consistent with reactivation from latency (Fig. 4A). In contrast, while latent genomes for pp71-deficient TR3 vector were measured in the same range as pp71-intact TR3, G-CSF treatment did not increase genome copy numbers suggesting that virus was unable to reactivate in the absence of pp71. Different from pp71-deficient TR3 we observed that the highly passaged laboratory strain AD169 was not able to establish latent infection even when containing an intact PC (Fig. 4B) suggesting that other genes deleted or mutated in AD169 are required for the establishment and/or maintenance of latent infection. We therefore interpret our results as evidence that pp71-deleted TR3 maintains the ability to establish latency but is limited in its ability to reactivate. This interpretation is consistent with observations reported for pp71-deficient HCMV in *in vitro* latency models^41,42^.

### Live-attenuated HCMV TR3 elicits effector memory T cell responses in non-human primates

We used humanized mice to monitor viral latency and reactivation *in vivo* since HCMV replication is highly species-specific. Therefore, animal models generally rely on studying the CMV of the respective animal species, e.g. RhCMV in rhesus macaques (RM) or MCMV in mice. However, it has been reported that a conditionally replicating, pentamer-intact AD169 (termed V160), elicited HCMV–specific T cell and antibody responses in RM^43,44^. Conditional replication was achieved by fusion of a degradation domain derived from the FK506-binding protein (FKBP) to the essential viral gene products IE1/2 and UL51 so that viral replication occurs only in the presence of a stabilizing ligand for FKBP. We previously reported that replication competent (pentamer-deficient) AD169 can infect RM fibroblasts, but does not produce viral progeny^45^. Similarly, we observed that HCMV TR3ΔUL78gag was able to infect RM fibroblasts resulting in low level viral and HIVgag protein expression, but did not generate infectious progeny (Fig. S3). These results suggest that even replication competent HCMV will be unable to disseminate in RM suggesting that RM cannot be used to monitor pathogenesis and attenuation of HCMV.

**Figure 5:**
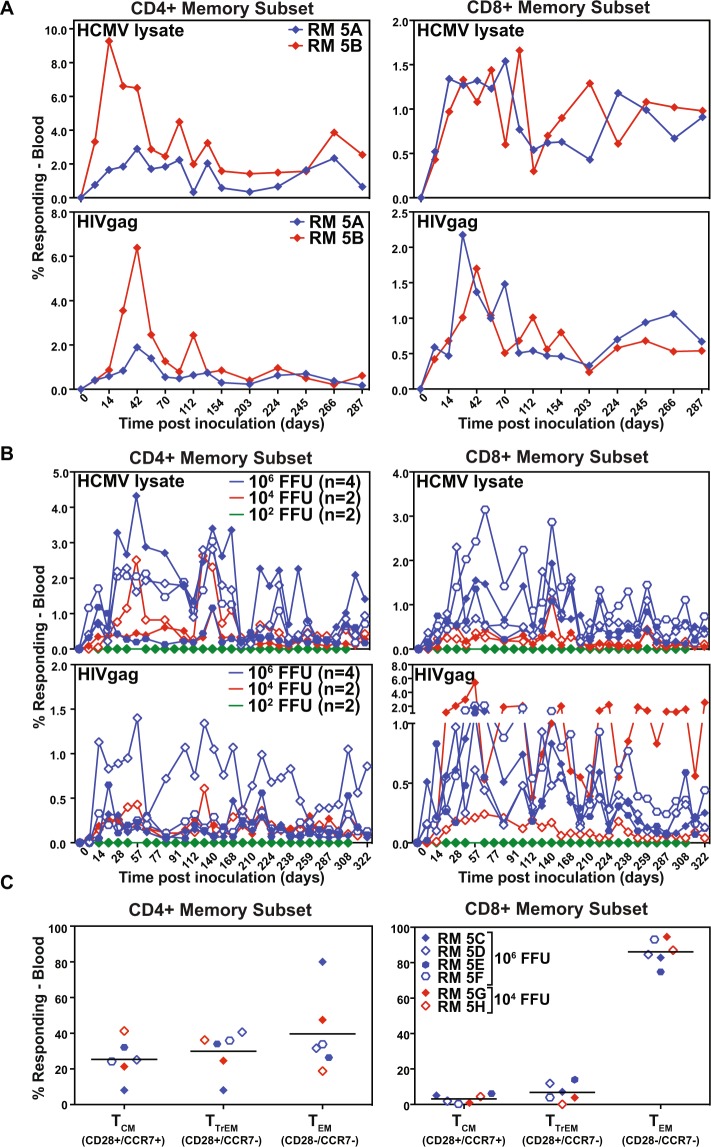
TR3-derived vectors elicit HIVgag-specific T_EM_ in RM. (**A**) TR3 elicits T cell responses to HIVgag in RM. Two RM were inoculated with 5 × 10^6^ FFU of TR3ΔUL78gag at day 0. PBMC were collected at the indicated days and HCMV-lysate as well as HIVgag-specific CD4+ and CD8+ T cell responses were measured by intracellular cytokine staining for TNFα and/or IFNγ. The frequency of responding T cells is shown as percent of total memory T cells. (**B**) pp71-deleted TR3 elicits T cell responses to HIVgag. RM were inoculated with 10^2^ FFU (n = 2), 10^4^ FFU (n = 2) or 10^6^ FFU (n = 4) of TR3ΔUL82gag at day 0 and HCMV lysate and HIVgag-specific T cell responses were determined as in A) at the indicated time points post-infection. (**C**) Frequency of memory populations within the HIVgag-specific CD4+ and CD8^+^ memory T cells in peripheral blood of the RM inoculated with 10^4^ FFU or 10^6^ FFU of TR3ΔUL82gag. Memory differentiation state was based on CD28 vs. CCR7 expression, delineating central memory (+/+T_CM_), transitional effector memory (+/− T_TrEM_), and effector memory (−/− T_EM_), as designated. The same colors and symbols are used in B) and in C) for the same animals.

However, despite this species-specificity, V160 was reported to elicit T cell responses to IE1/2, but not to the FKBP moiety^44^. Therefore, we sought to determine whether TR3-based vectors would be able to elicit T cell responses to inserted heterologous antigens in RM. Two RM were subcutaneously inoculated with 5 × 10^6^ FFU of TR3ΔUL78gag and monitored the CD4+ and CD8+ memory T cell response in peripheral blood mononuclear cells (PBMC) to HCMV lysate or to pooled overlapping 15mer peptides covering the entire HIVgag sequence by intracellular cytokine staining for TNFα and IFNγ. As shown in Fig. 5A, both RM elicited high frequencies of both CD4+ and CD8+ T cell responses to HCMV and HIVgag. Of note, there was no detectable HCMV-specific T cell response prior to inoculation despite the fact that all animals are naturally infected with RhCMV suggesting little or no shared T cell epitope sequences. Next, we determined whether pp71-deficient vectors would elicit HCMV and HIVgag-specific T cell responses and whether this ability would be vector dose dependent by inoculating 8 RM with 10^2^ FFU (n = 2), 10^4^ FFU (n = 2) or 10^6^ FFU (n = 4) of TR3ΔUL82gag. All RM developed robust HCMV-lysate and HIVgag-specific responses upon inoculation with TR3ΔUL82gag at 10^4^ and 10^6^ FFU, but not 10^2^ FFU (Fig. 5B). Phenotyping of the HIVgag responsive CD8+ T cells confirmed that the majority of T cells, particularly CD8+ T cells, elicited by the pp71-deficient vectors displayed a T_EM_ phenotype (Fig. 5C). Taken together these data demonstrate that despite an inability to replicate in RM cells, HCMV TR3 vectors were capable of eliciting robust effector memory T cell responses in RM to a heterologous antigen inserted into the viral genome at a subcutaneous dose as low at 10^4^ FFU.

### T cell immunogenicity of pentamer-deficient TR3

While the PC subunits UL128, UL130 and UL131 are dispensable for viral growth in fibroblasts, the glycoproteins gH and gL are essential since they form a trimeric complex with gO that binds to the cellular platelet-derived growth factor receptor α and this interaction is required for entry into fibroblasts^33,46^. Given the important role of the PC in viral tropism, HCMV strains that lack the non-essential subunits are likely attenuated *in vivo*. Indeed, strain 68-1 RhCMV that lacks PC subunits homologous to UL128 and UL130 shows decreased viremia and shedding upon primary infection of RM^47^. Interestingly however, despite lacking a functional PC, 68-1 RhCMV-based vaccines protect against HIV, TB and malaria^5–7^.

**Figure 6:**
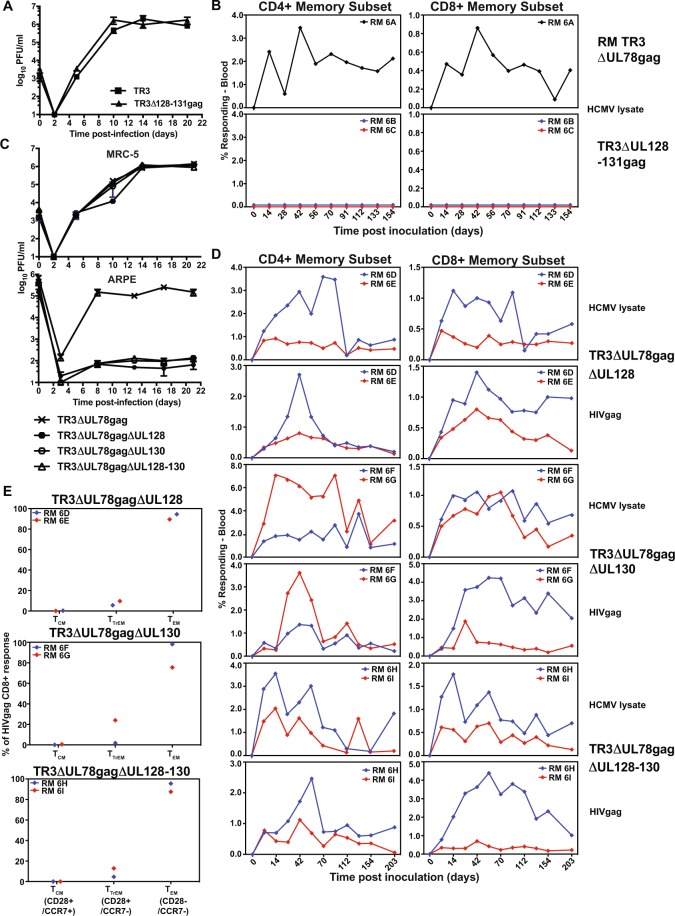
T cell responses to pentamer-deficient TR3 vectors in RM. (**A**) *In vitro* growth curve of TR3 and TR3ΔUL128-131gag. MRC-5 cells were inoculated with TR3 or TR3ΔUL128-131gag at a MOI = 0.01 and cell culture supernatants were collected at the indicated times. Virus was titered by plaque assay on MRC-5 cells. The average titer of triplicate experiments (+/− SD) is shown. (**B**) UL128-131-deleted TR3 does not elicit T cell responses in RM. Two RM were inoculated with 5 × 10^6^ FFU TR3ΔUL128-131gag, one RM was inoculated with 5 × 10^6^ FFU TR3ΔUL78gag and the T cell response to HCMV-lysate or HIVgag was measured at the indicated days. **(C**) *In vitro* growth curve of PC-deficient TR3. MRC-5 cells (top) or ARPE cells (bottom) were inoculated with the indicated viruses at MOI = 0.01 (MRC-5) or MOI = 5 (ARPE) and cell culture supernatants were collected at the indicated times. Virus was titered as above and the average titer of triplicate experiments (+/− SD) is shown. (**D**) HIVgag-specific T cell response elicited by PC-deficient TR3. RM (n = 2 per recombinant) were inoculated with 5 × 10^6^ FFU of the indicated PC-deficient vectors on day 0 and the HIVgag-specific or HCMV-lysate CD4+ and CD8+ memory T cell response frequencies were determined in PBMC by intracellular cytokine staining for TNFα and/or IFNγ using overlapping HIVgag peptide pools at the indicated days. (**E**) Frequency of memory populations within the HIVgag-specific CD8^+^ memory T cells in peripheral blood of the six RM in (**D**) inoculated with the indicated viruses. Memory differentiation state was based on CD28 vs. CCR7 expression, delineating central memory (+/+T_CM_), transitional effector memory (+/− T_TrEM_), and effector memory (−/− T_EM_), as designated.

To examine the role of the non-essential subunits UL128, UL130 or UL131 for TR3-based vector immunogenicity we initially deleted the entire coding regions for UL128, UL130, and UL131 by replacing the region with HIVgag (Fig. S3). As expected, TR3ΔUL128-131gag grew similar to parental TR3 in fibroblasts (Fig. 6A). However, when TR3ΔUL128-131gag was inoculated into RM, we did not observe HCMV-specific or HIVgag-specific T cell responses suggesting that this recombinant had lost the ability to infect RM (Fig. 6B). Since the homologue of UL131 is intact in 68-1 RhCMV whereas UL130 is partially and UL128 fully deleted^48^ we next evaluated whether deletion of UL128 and/or UL130 would similarly affect vector function. As expected, TR3ΔUL78gag additionally deleted for UL128, UL130 or UL128+ UL130 grew similar to TR3ΔUL78gag in fibroblasts, but not in epithelial cells (Fig. 6C). We next inoculated two RM with each PC-deficient vector and monitored the HIVgag and HCMV-specific CD4+ and CD8+ T cell response in PBMC by intracellular cytokine staining over time. As shown in Fig. 6D, all RM developed robust HIVgag- and HCMV-specific T cell responses despite PC deficiency. Moreover, the majority of the HIVgag-specific CD8+ T cells displayed a T_EM_ phenotype (Fig. 6E). Thus, PC-deficient TR3 maintained the ability to elicit T cell responses in RM. However, the UL131 gene product seems to act not only as a PC subunit but seems to provide an additional, as yet unknown, function that is essential for virus infectivity in RM and possibly humans.

### Attenuation of TR3 by the combined deletion of pentamer subunits and pp71

**Figure 7:**
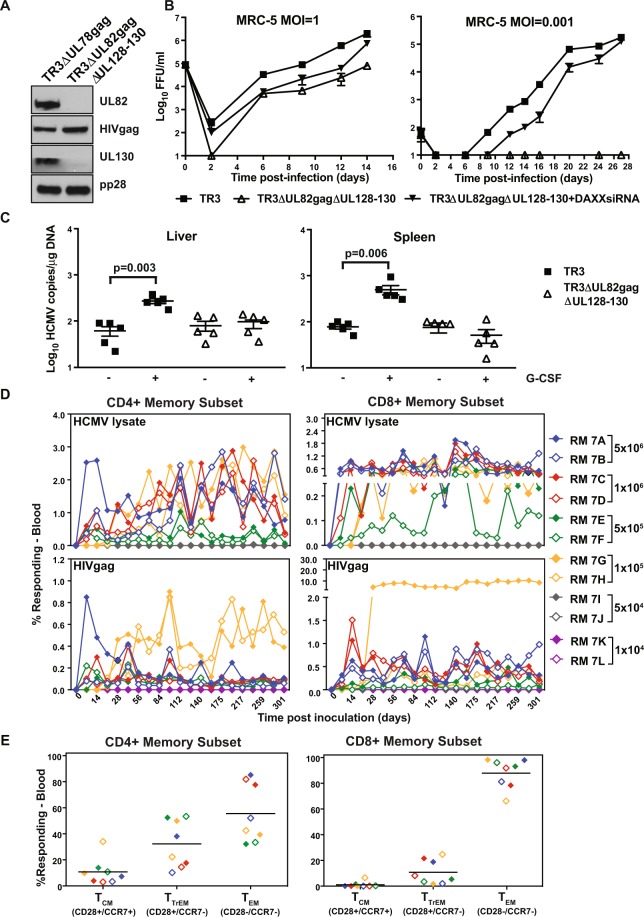
*In vitro* and *in vivo* characterization of pentamer and pp71-deficient TR3 vectors. (**A**) HIVgag expression and lack of pp71 and UL130 expression for TR3ΔUL82gagΔUL128-130. Lysates from MRC-5 cells infected with the indicated viruses (MOI = 0.5) for 96 h were electrophoretically separated and probed for the indicated proteins by immunoblot. The blots for each protein are shown as cropped from different parts of the same gel. **(B**) Viral growth in fibroblasts in the presence or absence of DAXX siRNA. MRC-5 cells were infected with the indicated viruses at MOI = 1 or 0.001 in the presence or absence of siRNA targeting DAXX. Virus titers in the supernatant were determined at the indicated days on BJ-pp71 cells. (**C**) Latency and reactivation in humanized mice. HuNSG mice were infected with the indicated viruses and treated with G-CSF as shown. Infections and determination of viral genome copy numbers as well as statistical analysis were as described in Fig. 4. (**D**) Immunogenicity in RM. RM were inoculated with 10^4^ FFU, 5 × 10^4^ FFU, 10^5^ FFU, 5 × 10^5^ FFU, 10^6^ FFU or 5 × 10^6^ FFU (n = 2 each dose) of TR3ΔUL82gagΔUL128-130 at day 0 and HIVgag-specific or HCMV-lysate T cell responses were determined as described in Fig. 5. The frequency of responding T cells is shown as percent of total memory T cells. E) Frequency of memory populations within the HIVgag-specific CD4+ and CD8+ memory T cells in peripheral blood of all RM in (**D**) demonstrating HIVgag-specific T cell responses (≥1 × 10^5^ FFU inoculum). Memory differentiation state was based on CD28 vs. CCR7 expression, delineating central memory (+/+T_CM_), transitional effector memory (+/− T_TrEM_), and effector memory (−/− T_EM_), as designated. The same colors and symbols are used in (**D**) and in (**E**) for the same animals.

Since the limited tissue tropism of a PC-deficient vector would likely further increase the safety profile of vectors that are live-attenuated by deletion or inactivation of pp71 we wanted to determine the impact of a combined PC and pp71-deletion on cell-tropism, viral latency and cellular immune responses. Therefore, we deleted UL128 and UL130 from TR3ΔUL82gag using BAC mutagenesis. The deletion was confirmed by PCR analysis and NGS (Fig. S4). Upon reconstitution of virus in the presence of DAXX siRNA we confirmed loss of UL130 and pp71 by immunoblot of infected cell lysates (Fig. 7A) and demonstrated that virus growth in MRC-5 fibroblasts was impaired at low MOI unless cells were transfected with DAXX siRNA (Fig. 7B). As expected, the PC and pp71-deficient TR3 vector did not grow in non-fibroblast cells even at high MOI (Fig. S5). To determine whether the combined pp71 and PC deficiency affected the ability of TR3 to establish latency we inoculated humanized mice either with unmodified TR3 or with TR3ΔUL82gagΔUL128-130 and measured genome copy numbers in spleen and liver in the presence or absence of G-CSF. In the absence of G-CSF, similar amounts of latent viral genomes were detected for both TR3 and the PC-deficient recombinant (Fig. 7C). However, upon G-CSF treatment only TR3 displayed elevated viral genome copies consistent with viral reactivation, whereas TR3ΔUL82gagΔUL128-130 remained latent similar to the pp71-deleted parental recombinant. Taken together these data suggest that, while the PC is required for infection of non-fibroblast cells with cell-free virus *in vitro*, this complex does not seem to be essential for the transfer of virus from infected fibroblasts into human myeloid-lineage cells *in vivo*. Furthermore, once these cells are infected, the PC does not impact the establishment of latency. The lack of reactivation in response to G-CSF treatment is likely primarily due to lack of pp71.

To determine whether PC and pp71-deleted TR3 vectors retained the ability to elicit T cell responses to HIVgag we inoculated RM with different doses of TR3ΔUL82gagΔUL128-130 and monitored both HIVgag-specific and HCMV-specific T cell responses. As shown in Fig. 7D, we did not detect HIVgag-specific or HCMV-specific T cell responses in RM inoculated with PC and pp71-deficient TR3 at 10^4^ and 5 × 10^4^ FFU. However, all RM developed both HCMV and HIV-gag-specific T cell responses above a dose of 10^5^ FFU, although T cell responses were detectable about one week earlier in animals that received higher doses (Fig. 7D). Compared to pp71-intact TR3 it seems that deletion of the PC-subunits decreased the ability of pp71-deficient TR3 to elicit T cell responses by about 1 log. However, above a threshold of approximately 10^5^ FFU the resulting live attenuated vector was able to elicit and maintain effector memory T cell responses to the inserted heterologous antigen (Fig. 7E).

To determine whether the TR3 vectors elicited antibodies we performed enzyme linked immunosorbent assays (ELISAs) using lysates of HCMV-infected cells as antigens. We examined RM inoculated with TR3ΔUL78gag and increasing doses of TR3ΔUL82gag or TR3ΔUL82gagΔUL1280-130 as shown above. All animals that developed T cell responses to HIVgag and HCMV also showed above-background HCMV-reactive antibodies that peaked around day 42 post-inoculation (Fig. S6). However, unlike the T cell responses, which remained stable, the antibody response waned over time consistent with a lack of viral replication in the animals. Together these data suggest that similar to our observations in RhCMV, TR3 represents a robust T cell-inducing viral vector platform, even when highly attenuated, whereas the ability to elicit antibodies is limited, at least at the relatively low doses used here.

## Discussion

As a first step towards achieving the goal of clinical testing of HCMV vectors we designed, constructed and characterized a novel live-attenuated HCMV-based vaccine platform. The attenuation strategy was derived from extensive studies in the RhCMV model demonstrating that deletion of the gene encoding the tegument protein pp71 rendered vectors deficient for inter- and intra-host spreading while maintaining all of the desired immunological characteristics as well as protection against SIV challenge^12,13^. Since pp71 counteracts host transcriptional co-repressors of the promyelocytic leukemia protein (PML)-nuclear body complex^49^ (sometimes referred to as “intrinsic” immunity^24^), tumor suppressors^50^ as well as innate immune signalling cascades^51^, this strategy thus engages several conserved antiviral host mechanisms to limit viral lytic replication. Deletion of pp71 as our primary attenuation strategy thus limits, if not completely eliminates, the possibility of HCMV pathogenesis in immunocompromised individuals which requires lytic replication for viral dissemination.

Since pp71 facilitates the expression of IE proteins which in turn activate viral gene expression and genome replication, it is considered a key protein in deciding establishment of latency versus activation of the lytic transcriptional cascade^42^. Similar to other tegument proteins, pp71 is delivered into the cytosol during viral entry. However, while pp71 travels to the nucleus in cells that are permissive for lytic replication, it remains cytoplasmic in incompletely differentiated myeloid cells as well as CD34+ hematopoietic stem cells resulting in lack of IE expression and viral latency *in vitro*^52^. Thus, by silencing IE expression cellular intrinsic defence proteins likely play a key role in the establishment and maintenance of HCMV latency. Consistent with this hypothesis we observed that pp71-deleted TR3 was able to establish latency in human CD34+ cells but could not be reactivated upon G-CSF treatment. In addition, it is possible that viral reactivation was further inhibited because deletion of the UL82 gene also eliminates the latent undefined nuclear antigen (LUNA) encoded on the opposite strand of UL82, a feature that is not conserved in the RhCMV homologue Rh110^53^. LUNA was recently shown to de-sumoylate PML thus dispersing PML-bodies, a process that seems to be important for viral reactivation in response to external stimuli^54^. Thus, UL82/LUNA-deleted TR3 preferentially establishes latency over lytic replication and is deficient in its ability to reactivate from latency.

The ability of pp71-deleted TR3 to persist *in vivo* is one of the desired features for a HCMV-based vector platform since viral persistence enables the maintenance of T_EM_ that require repeated antigen exposure. In our prototype vectors, the HIVgag antigen was expressed by replacing the coding regions of UL78 and UL82 which are expressed with early to late kinetics *in vitro*^55^. Since early and late genes require transcriptional activation by viral IE protein, HIVgag would not be expected to be expressed during viral latency. Nevertheless, we observed robust and lasting HIVgag and HCMV-specific T_EM_ responses in RM inoculated with as little as 10^4^ FFU suggesting that some viral and heterologous insert gene expression occurs despite limited IE expression, perhaps as a consequence of partial reactivation. Thus, similar to our observation in RhCMV, we conclude that replacement of UL82 with a given transgene provides sufficient amount of antigen to elicit and maintain T_EM_. The UL82-replacement design has the additional advantage to eliminate the possibility of reversal of UL82-deletion by homologous recombination with endogenous HCMV in chronically infected individuals. Although recombination with endogenous virus is likely a very rare event in immunocompetent individuals, as we have documented for pp71-deleted in RhCMV^12^, there is clear evidence for recombination between different HCMV strains in highly viremic immunosuppressed individuals under the selective pressure of antiviral drugs^56^. Since repair of UL82 by homologous recombination would result in an antigen-less TR3, the net result of homologous recombination would be infection by an additional HCMV variant in individuals that already carry HCMV, similar to the frequently observed superinfection with HCMV^57^.

In addition to pp71-deletion we explored attenuation by deletion of the UL128, UL130 and UL131 subunits of PC. Unlike gH/gL, these subunits are non-essential for growth *in vitro* and, in fact, are often spontaneously deleted upon propagation of clinical isolates in fibroblasts^58^. However, different from other clinical isolates, the TR3 PC seems to be stable upon repeated passages as demonstrated by NGS of passage 20 of TR3ΔUL82gag and TR3. This inherent stability is possibly due to the ability of HCMV TR to generate relatively high amounts of cell free virus which reduces the selection pressure for PC-deficient HCMV^59^. Nevertheless, the PC is clearly functional *in vitro*, as demonstrated by increased infection of non-fibroblast cells, as well as *in vivo*, since PC-intact TR3 elicited T cell responses in RM at a lower threshold compared to PC-deleted TR3. Surprisingly, we observed that deletion of all three subunits resulted in vectors that had lost the ability to elicit T cell responses in RM even at the highest dose tested whereas deletion of UL128 and/or UL130 only increased the dose required to elicit T cell responses. Since all of these mutants are PC-deficient, this result suggests a PC-independent role for UL131 in promoting infection. At present this function is unknown. However, this observation is reminiscent of our previous finding that the NK cell evasion protein Rh159 of RhCMV is non-essential for growth *in vitro*, but required for infection *in vivo*^60^ suggesting that UL131 might contribute to viral evasion of innate immune control. Further studies will be required to elucidate this unexpected, PC-independent function of UL131.

A second important rationale for pentamer deletion is our observation that RhCMV vectors derived from strain 68-1 elicit CD8+ T cells recognizing peptides in the context of major histocompatibility complex (MHC) class II or the non-polymorphic, highly conserved MHC class Ib molecule MHC-E instead of classical, polymorphic MHC-Ia molecules^61,62^. In contrast, wildtype RhCMV or vectors based on strain 68-1.2 in which the RhCMV homologs of UL128 and UL130 were reinserted into 68-1, elicit CD8+ T cells restricted by MHC-Ia. Thus, it will be important to evaluate both PC-intact and PC-deleted HCMV-vectors in clinical trials to determine whether this “immune programming” can be translated from RM to human. The data presented here suggest that it will be possible to evaluate PC-deficient HCMV that is additionally attenuated by pp71-deletion although it might require a higher dose.

Most previously reported clinical studies with attenuated HCMV involved PC-deficient HCMV strains^16,63,64^. In all of these cases only very modest T cell responses that did not show the CMV-typical T_EM_ bias were observed^63,65,66^. The lack of T_EM_ bias indicates an inability for persistent antigen presentation due to a general inability to establish a persistent infection and to repeatedly provide viral antigens to professional antigen presenting cells. It is possible that this lack of T_EM_ bias was a consequence of PC-deficiency. However, we observed robust T_EM_ responses elicited and maintained by PC-deficient TR3 in RM, even upon additional attenuation by pp71-deletion. Similarly, we reported extensively on the robust T cell responses elicited by PC-deficient RhCMV to heterologous antigens inserted into strain 68-1^6,7,67^. However, in addition to PC-deficiency, all previous clinical studies relied on HCMV strains and clones that display multiple genetic alterations as a results of passaging in tissue culture, including the low passage isolate Toledo^30^. Our data thus suggest that the lack of PC was likely not the main reason for the lack of robust and durable T_EM_ responses (as well as unconventionally restricted CD8+ T cells^66^) in previous clinical trials. This conclusion is supported by the finding that even a PC-intact version of the laboratory strain AD169 was unable to establish latency in humanized mice. Our strategy of using a wildtype-like vector backbone that is attenuated by a very specific molecular mechanism and contains a heterologous antigen will thus allow us to specifically address the role of the PC in the elicitation and maintenance of T cell responses, including unconventionally restricted CD8+ T cells.

One of the challenges of manufacturing viral vaccines that are attenuated by deletion of essential genes is the need for a production cell line that is compatible with vaccine manufacturing procedures. Generally, this is done by providing the deleted viral gene product *in trans* either by transient transfection or by generating a stable complementing cell line^68^. While these approaches are well-established for transformed cell lines, efficient growth of HCMV only occurs in primary human cells which are difficult to transfect transiently with high efficiency and it is even more difficult to generate cloned, immortalized cell lines that maintain high levels of viral transgene expression. One possible strategy to overcome this limitation is to generate conditionally replicating HCMV by fusion of a degradation domain to essential viral proteins which allows the production of virus in primary cells upon addition of a stabilizing agent^44^. However, since the long-term maintenance of T_EM_ responses likely requires the replication of viral genomes, at least at a low level, this approach runs the risk of eventually selecting for revertants *in vivo* upon viral genome replication. We demonstrate here an alternative production method that relies on siRNA-mediated suppression of anti-viral host cell proteins thus eliminating the need for the viral protein to support viral growth *in vitro*. Specifically, we show that by eliminating DAXX expression we can achieve viral titers for UL82-deleted TR3 that approach those of UL82-intact TR3. This approach is generally applicable to viral gene products whose main function is to counteract an anti-viral host cell protein. Importantly siRNA mediated DAXX knockdown was highly efficient in primary human cell lines, including MRC-5 cells which are approved for the manufacturing of viral vaccines for human use. Recently, this manufacturing strategy was used in the development of improved formulations for TR3ΔUL82gagΔUL1280-130 with respect to freeze-thaw characteristics and short-term liquid stability^69^.

In sum, the TR3-based live attenuated HCMV vaccine vector platform not only maintains key immunological characteristics such as the ability to elicit lasting T_EM_ to an inserted heterologous antigen, but is also stable over many passages and can be manufactured in approved cell lines. As such, this vector platform is ideally suited to evaluate the unique aspects of CMV-based immunity in humans.

## Material and Methods

### Cell lines

Fetal human lung fibroblasts (MRC-5) were a gift from the international AIDS vaccine initiative (IAVI), human umbilical vein endothelial cells (HUVEC) were purchased from Lonza (cc-2519), human retinal pigmented epithelial cells (ARPE-19) and human Astrocytoma cell line (CCF-STTG1) were obtained from ATCC (CRL-2302 and CRL-1718). Primary rhesus fibroblasts (RF) were generated as described^70^. MRC-5, ARPE-19 and RF were grown as monolayers in Dulbecco’s modified Eagle’s medium (DMEM) (Mediatech) supplemented with 10% fetal bovine serum (FBS) (HyClone), 4.5 g/l glucose, L-glutamine and sodium pyruvate, and antibiotics [penicillin (10 units/ml) and streptomycin (10 μg/ml)]. HUVEC were cultured in endothelial growth medium EGM-2 (Clonetics) containing 2% FBS, human recombinant vascular endothelial growth factor, basic fibroblast growth factor, human epidermal growth factor, insulin-like growth factor-1, hydrocortisone, ascorbic acid, gentamicin and amphotericin B (1 μg/ml each) (high-serum medium). CCF-STTG1 were grown in RPMI-1640 (HyClone) medium supplemented with 10% FBS, 4.5 g/l glucose, L-glutamine and sodium pyruvate, and antibiotics [penicillin (10 units/ml) and streptomycin (10 μg/ml)].

Primary human foreskin fibroblasts immortalized with telomerase (BJ-5ta) were described previously^28^ and were purchased from ATCC (CRL-4001). BJ-5ta cells were grown in four parts DMEM, containing 4 mM L-glutamine, 4.5 g/l glucose and 1.5 g/l sodium bicarbonate, and one part of Medium 199 (Corning), supplemented with 10 µg/ml hygromycin B to maintain the telomerase expression, and 10% FBS.

To generate BJ-pp71 cells, BJ-5ta cells were first transduced with pLVX-blast (Clontech) into which the pp71-encoding UL82 gene (obtained by PCR from HCMV AD169) was inserted under control of a tetracycline-regulated-transactivator (tTa)-dependent promoter. Transductants were selected with 5 µg/ml blasticidin followed by a second transduction of pLVX-Tet-On-Advanced (Clontech), harboring the tTa and the neomycin gene which allows for selection by 100 µg/ml G418. Pp71 expression is inducible by the addition of 1 µg/ml doxycycline (DOX). All cells were tested for mycoplasma contamination using Universal Mycoplasma Detection Kit (ATCC 30-1012K^TM^).

### HCMV

HCMV AD169 and AD169rUL131, both of which express GFP in the UL21.5 locus, were described previously^71^ and were kindly provided by Dr. Tom Shenk, Princeton University. The HCMV isolate TR was isolated from an AIDS patient as previously reported reported^72^. TR-BAC (GenBank Accession Number: AC146906.1) was cloned by substituting non-excisable BAC DNA into the US2-6 region of the HCMV TR isolate as described^16^. TR-GFP was generated by inserting a GFP expression cassette into US7 together with a loxP site downstream of the BAC cassette and the US2-US7 genes from AD169 together with a second loxP site upstream of the BAC-cassette as described previously previously^18^. The BAC of TR-GFP was kindly provided by Dong Yu, Washington University.

TR3 was derived from TR-GFP by introducing UL97 from HCMV strain AD169 to restore ganciclovir sensitivity and by making the BAC cassette self-excising in human fibroblasts by inserting *Cre* via Lambda (λ) Red-mediated homologous recombination^20^. In the first step we replaced the UL97 ORF with a galactokinase (galK) and kanamycin (Kan) selection cassette generated by PCR using primers containing 50 bp homology to regions flanking the UL97 ORF. *E. coli* strain SW105 containing the BAC of TR-GFP was electroporated with the PCR product for recombination and selected for the presence of galK on M63 minimal plates containing galactose. Next, we generated a recombination fragment that contained AD169 UL97 flanked by a DNA sequence homologous to flanking regions of TR UL97. This DNA fragment was then used to replace the GalK/Kan selection cassette with the AD169 UL97 by homologous recombination through selecting for loss of galK on 2-deoxy-galactose (DOG) plates. The resulting BAC construct was analyzed by restriction digest and the UL97 sequence was verified by sequencing of the corresponding PCR product. To generate a self-excising BAC cassette we inserted the *Cre* recombinase gene under control of the SV40 promoter downstream of the chloramphenicol resistance gene into the BAC cassette. Using a series of molecular cloning steps we generated a recombination fragment that contained the SV40/*Cre* expression and the galK/Kan resistance cassettes flanked upstream by a DNA sequence homologous to the BAC cassette and downstream by a loxP site and a DNA sequence homologous to a gene region downstream of the GFP gene. This DNA fragment was inserted into UL97-repaired TR-GFP using homologous recombination and selection for Kan resistance. In the next step, the galK/Kan selection cassette was removed by homologous recombination with a 100 bp double stranded DNA fragment with 50 bp homology to flanking regions. The resulting recombinant HCMV TR3 was then counter-selected on DOG plates for loss of galK. The final TR3 construct was analyzed by restriction digest, PCR and next generation sequencing (NGS) (GenBank Accession number: MN075802).

Recombinant HIVgag expressing vectors were constructed as follows: The HIVgag insert (GenBank Accession number: MN193852) was sub-cloned from a multigene construct containing the gag, reverse transcriptase, integrase and nef (GRIN) sequences of an HIV-1 subtype A isolate^73^, (kindly provided by IAVI). TR3ΔUL78gag was generated by homologous recombination in *E. coli* strain EL250 containing heat-inducible λ red genes and an arabinose-inducible FLP recombinase^74^. Using primers containing 50 bp homology to regions flanking UL78, HIVgag was amplified by PCR together with a Kan resistance cassette flanked by FRT sites from plasmid pCP015. EL250 bacteria containing TR3 BAC were electroporated with the PCR product for recombination. Recombinants were selected for Kan resistance, followed by selection of FRT-mediated loss of Kan upon arabinose induction of FLP recombinase.

TR3ΔUL82gag was generated using the galK/Kan two-step recombination and selection system. In step 1 UL82 was replaced by homologous recombination in TR3-BAC-containing SW105 bacteria with a PCR product containing a galK/Kan expression cassette with 50 bp flanking homology to UL82. Upon positive selection for Kan and chloramphenicol, UL82 replacement was confirmed by restriction digest and diagnostic PCR. In step 2 the galK/Kan cassette was replaced with a PCR product containing the HIVgag insert and the same flanking homology arms. Recombinants were selected on DOG and chloramphenicol minimal media with glycerol as the carbon source. Using the galK/Kan selection method we introduced deletions of UL128 and/or UL130 into TR3ΔUL78gag or TR3ΔUL82gag to generate TR3ΔUL78gagΔUL128, TR3ΔUL78gagΔUL130, and TR3ΔUL78gagΔUL128-130 or TR3ΔUL82gagΔUL128-130. To generate TR3ΔUL128-131gag we used galK/Kan to replace the entire gene region spanning UL131, UL130 and UL128 with the HIVgag ORF. All BAC constructs were verified by NGS.

Viruses were generated by electroporation of the BAC DNA into MRC-5 cells. TR3ΔUL82 was recovered in the presence of 5 nM DAXX siRNA (duplex of 5′-GCUACAAGCUGGAGAAUGAUU-3′ and 3′-UUCGAUGUUCGACCUCUUACU-5′) (Dharmacon). Viral stocks were prepared in MRC-5 cells cultured in DMEM with 10% FBS upon transfection of 5 nM DAXX siRNA with Lipofectamine 2000 (Invitrogen) 24 h prior to infection with an MOI = 0.01. DAXX siRNA was re-transfected on day 10 and, at full cytopathic effect (CPE), the supernatant from infected cells was harvested and purified on a 20% sorbitol cushion. Purified virus was resuspended in DMEM with 2% FBS and stored at −80 °C.

### Next generation sequencing

BAC DNA was prepared using the NucleoBond PC 100 kit (Macherey-Nagel) following the manufacturer’s instructions. To generate purified viral DNA for NGS, we used a modified extraction protocol originally designed for the extraction of Polyoma virus DNA from mouse cells^75^. Viral supernatants were harvested at full CPE, cellular contaminants and residual cells were removed by centrifugation initially at 2,000 × g for 10 min at 4 °C and subsequently at 7,500 × g for 15 min. The virus was pelleted from the clarified medium by overlaying a sorbitol cushion (20% D-sorbitol, 50 mM Tris [pH 7.4], 1 mM MgCl_2_) and centrifuging at 82,000 × g for 1 h at 4 °C in a Beckman SW28 rotor. The generated pellet was resuspended in 500 μl 10.1 TE Buffer (10 mM Tris, pH 8.0; 0.1 mM EDTA, pH 8.0) and 500 μl 2x lysis buffer (20 mM Tris-Cl, pH 8.0; 50 mM EDTA, pH 8.0; 200 mM NaCl; 1.2% w/v SDS) was added. Finally, to digest the purified virions, 250 μg Proteinase K was added and the solution was incubated for 2 h at 37 °C. The viral DNA was phenol/chloroform extracted twice and precipitated with absolute ethanol at −80 °C overnight. The DNA was pelleted for 20 min at 13,200 × g at 4 °C, washed once with 70% ethanol, and subsequently resuspended in DNase-free water. DNA concentrations were determined using a ND-1000 Spectrophotometer (NanoDrop Technologies).

Illumina sequencing libraries were generated as previously described^76^. Briefly, DNA was fragmented using an S2 Sonicator and was then converted to libraries using the standard TruSeq protocol. Libraries were examined on a Bioanalyzer (Agilent) and the concentration was determined using real time PCR and SYBR Green fluorescence. NGS was performed using a MiSeq NGS System (Illumina). Libraries were loaded into a MiSeq reagent cartridge at a concentration of 9 pM and single read sequencing was performed for 300 cycles with 6 additional cycles of index reads. The resulting data was imported into Geneious and the sequencing reads were trimmed of all regions exceeding the error probability limit of 0.1% to minimize sequencing errors. All reads with a total length of fewer than 50 bp after quality control were eliminated from further analysis to increase the likelihood of specific alignments during *de novo* and reference guided assemblies. Viral genomes were first *de novo* assembled using the processed sequencing data, and subsequently all reads were aligned to the generated consensus sequence in a reference guided assembly to examine potential SNPs.

### Titration of pp71-deleted TR3

BJ-pp71 cells were seeded at approximately 12,000 cells/well into 96-well plates. The next day, viral supernatants were diluted in warm media (DMEM supplemented with 10% heat-inactivated tetracycline free FBS (Atlanta Biologicals), 1% 100X Pen-Strep-Glutamate (Invitrogen) and 1 µg/ml doxycycline). The cells were infected with serial virus dilutions and incubated for ~18 hours. Methanol (at −20 °C) was gently added to the side of each well. Plates were sealed and placed at −20 °C for 10–20 min followed by removal of the methanol. Using 200 to 300 μl of wash buffer (Dulbecco’s phosphate buffered saline (DPBS) with 0.05% Tween), plates were washed 3 times for 5 min. 100 μl of blocking solution (DPBS with 10% FBS) was added per well and incubated at 37 °C for 60 min. The blocking solution was removed and 50 μl of primary antibody solution (1:1000 mouse anti-HCMV (0898) antibody (Santa Cruz, Catalog No. sc-58118) in blocking solution) was added followed by incubation at 37 °C for 60 min. Using 200 to 300 μl of wash buffer per well the plates were washed 3 × 5 min. and 50 µl of the secondary antibody solution (1:1000 goat anti-mouse horseradish peroxidase antibody (Santa Cruz, Catalog No. sc-2005) in blocking buffer) was added to each well followed by incubation at 37 °C for 60 min. Plates were washed 3x for 5 min using 200 to 300 μl of wash buffer per well. Wash buffer was carefully removed after the final wash. 50 µl of TrueBlue Stain (KPL) was added to each well. Staining was monitored by microscopy and, once developed, the staining solution was removed and discarded. Each well was washed twice with 200 μl of deionized water to stop the reaction. Images were captured for each well and number of distinct blue foci were counted. All dilutions in the quantifiable range (1 to 200 foci/well) were counted by fluorescent microscopy, and for analysis of linearity, each dilution’s titers were independently calculated. The concentration of focus-forming units (FFU/ml) were calculated by the following formula: FFU/ml = foci count × dilution factor × 10.

### Growth curves

For each time point, cells (MRC-5, HUVEC, ARPE-19, or CCF-STTG1) were plated separately in triplicate into 24-well plates. For each cell type, the media used at this step was the same as the cell media used for that cell type’s growth and propagation. After 24 h, each cell type was infected with recombinant HCMV at the indicated MOI. After 2 h, the inoculum was removed, cells were washed 3 times with phosphate buffered saline (PBS), and fresh media was added. Supernatants were collected at the indicated time after the relevant HCMV infection and either frozen at approximately −80 °C or titrated on BJ-pp71 cells.

### Immunoblotting

Total proteins were extracted from HCMV infected fibroblasts using Cell Lysis Buffer (Cell Signaling Technologies). Extracted proteins were quantified by the BCA method (Pierce BCA Protein Assay kit, Thermo Scientific) and diluted to the same protein concentrations. Equivalent amounts of each group were separated on 8–12% SDS polyacrylamide gels. Proteins were transferred to Immobilon-P Transfer Membranes and probed with the following specific antibodies: rabbit polyclonal immediate-early (IE)-specific antibody^77^; mouse monoclonal anti HCMV UL44 (CH16; Virusys Corporation), mouse monoclonal anti HCMV UL99 (pp28)^78^, mouse monoclonal anti HCMV UL82 (pp71))^79^ (kindly provided by Dr. Tom Shenk), rabbit polyclonal HIV-1 SF2 HIVgag p24 (#4250, NIH AIDS Reagent Program), mouse monoclonal anti-actin (MA5-11869; Thermo Scientific), rabbit polyclonal anti HCMV US6^22^, rabbit polyclonal anti HCMV UL130^32^ (kindly provided by David Johnson), rabbit anti DAXX (D7810, Sigma). Secondary antibodies: goat anti-mouse IgG-HRP (sc-2005; Santa Cruz) and goat anti-rabbit IgG-HRP (111-035-003; Jackson Immunoresearch). Membranes were stained multiple times after been stripped with Restore Western Blot Stripping buffer (Thermo Scientific).

### Engraftment and infection of humanized mice

All animal experiments were conducted under the approved Oregon Health and Science University (OHSU) Institutional Animal Care and Use Committee (IACUC) protocol (3498). All mice in this study were managed in accordance with the NIH Office of Laboratory Animal Welfare: “PHS Policy on the Humane Care and Use of Research Animals” and the recommendations of the American Association for Accreditation of Laboratory Animal Care (AAALAC): “The Guide for the Care and Use of Laboratory Animals, 8^th^ edition”. NOD-*scid*IL2Rγc null (NSG) mice were purchased from Jackson Laboratories and bred in-house. All mice were housed in micro-isolator cages in a designated specific pathogen-free facility at OHSU and fed sterile food and water. Mice were euthanized via CO_2_ administration according to AAALAC euthanasia guidelines. Engraftment of NSG mice with human CD34+ hematopoietic stem cells was performed as described previously^39^. These huNSG mice were infected with HCMV after pre-treatment with 1 mL of 4% Thioglycollate (Brewer’s Media; BD) by intraperitoneal (IP) injection to recruit monocyte/macrophages. At 24 h post-treatment, humanized mice were infected with HCMV-infected fibroblasts from two T150 flasks (approximately 10^5^ FFU of cell-associated virus per mouse) via IP injection. To promote HCMV reactivation, mice were treated with G-CSF and AMD3100 as previously described^39^. Briefly, G-CSF (100 µL at 300 µg/mL; Amgen) was delivered via an osmotic pump (1007D; Azlet) surgically implanted in the subcutaneous space beneath the dorsal skin on the mouse for 7 days. Additionally, on the same day as pump implantation, mice were also given a single IP injection of 125 µg of AMD3100 (1,1′-[1,4-Phenylenebis(methylene)]bis-1,4,8,11-tetraazacyclotetradecane octahydrochloride (Sigma).

### Quantitative PCR to determine HCMV genome copy numbers

Total DNA was extracted from approximately 1 mm^2^ sections of mouse spleen or liver using the DNAzol reagent (Life Technologies). Quantitative PCR (TaqMan) was performed on 1 µg of total DNA using TaqMan FastAdvance PCR Master Mix (Applied Biosystems), according to the manufacturer’s instructions. Primers and a probe recognizing HCMV UL141 were used to quantify HCMV genomes (probe: 5′CGAGGGAGAGCAAGTT; forward primer: 5′GATGTGGGCCGAGAATTATGA and reverse primer: 5′ATGGGCCAGGAGTGTGTCA). The probe contains a 5′ FAM reporter molecule and a 3′ quencher molecule (Applied Biosystems). The reaction was activated at 95 °C for 10 minutes followed by 40 cycles (15 s at 95 °C and 1 minute at 60 °C) using a StepOnePlus TaqMan PCR machine. Results were analyzed using ABI StepOne software (Applied Biosystems).

### T cell responses to TR3 in rhesus macaques

A total of 30 purpose-bred male or female rhesus macaques (RM) (*Macaca mulatta*) of Indian genetic background were used in this study. All RM were classified as specific pathogen free (SPF) as defined by being free of cercopithicine herpesvirus 1, D-type simian retrovirus, simian T-lymphotrophic virus type 1, SIV, and Mycobacterium tuberculosis, but were naturally infected with RhCMV. All RM used in this study were housed at the Oregon National Primate Research Center (ONPRC) in Animal Biosafety level (ABSL)-2 rooms. RM care and all experimental protocols and procedures were approved by the ONPRC IACUC. The ONPRC is a Category I facility. The Laboratory Animal Care and Use Program at the ONPRC is fully accredited by AAALAC and has an approved assurance number (#A3304-01) for the care and use of animals on file with the NIH Office for Protection from Research Risks. The IACUC adheres to national guidelines established in the Animal Welfare Act (7 U.S.C. Sections 2131–2159) and the Guide for the Care and Use of Laboratory Animals (8th Edition) as mandated by the U.S. Public Health Service Policy.

RM were inoculated sub-cutaneously with 10^3^-5 × 10^6^ FFU of TR3-derived recombinant HCMV expressing HIVgag. To monitor T cell and antibody responses, animals were subjected to bi-weekly blood draws and serum as well as PBMC were recovered. CD4+ and CD8+ T cell responses were measured in PBMC by intracellular cytokine staining^4,19,67^. For HIVgag-specific responses, PBMC were incubated with consecutive 15mer peptide mixes (11 amino acid overlap) comprising the inserted HIVgag sequence. For HCMV lysates, human fibroblasts infected with HCMV TR were scraped and pelleted to collect the cells at the time of maximum cytopathic effect. The cell pellet was resuspended and lysed by three cycles of freeze thaw and clarified by centrifugation at 3,000 × g for 10 min. Clarified supernatants were pelleted by ultracentrifugation through a 10% sorbitol cushion at 22,000 rpm in a Beckman SW32 rotor to concentrate the virus. The virus pellet was resuspended in the clarified cell lysate and mixed by vortexing. The mixture was aliquoted and frozen at −80 °C prior to use in the T cell assay.

For intracellular cytokine staining, the co-stimulatory molecules CD28 and CD49d (BD Biosciences) were added for 1 h, followed by addition of Brefeldin A (Sigma-Aldrich) for an additional 8 h. Co-stimulation without peptides served as background control. Stimulated cells were fixed, permeabilized and stained^4,19,67^ using combinations of the following fluorochrome-conjugated mAbs: SP34-2 (CD3; Pacific Blue, Alexa700), L200 (CD4; AmCyan, BV510), SK-1 (CD8α; PerCP-Cy5.5), MAB11 (TNFα; FITC, PE), B27 (IFNγ; APC), FN50 (CD69; PE-TexasRed), B56 (Ki-67; FITC), and in polycytokine analyses, JES6-5H4 (IL2; PE Cy-7). Data was collected on an LSR-II (BD Biosciences). Analysis was performed using FlowJo software (Tree Star). Lymphocytes were gated for CD3+ and progressive gating on CD4+ and CD8+ T cell subsets. Antigen-responding cells in both CD4+ and CD8+ T cell populations were determined by their intracellular expression of CD69 and one or more cytokines. After subtracting background, the raw response frequencies were memory corrected^4,19,67^ using combinations of the following mAbs to define the memory vs. naïve subsets: SP34-2 (CD3; Alexa700, PerCP-Cy5.5), L200 (CD4; AmCyan), SK-1 (CD8α; APC, PerCP-Cy-5.5), MAB11 (TNFα; FITC), B27 (IFNγ; APC), FN50 (CD69; PE), CD28.2 (CD28; PE-TexasRed), DX2 (CD95; PE), 15053 (CCR7; Pacific Blue), and B56 (Ki-67; FITC). For memory phenotype and polycytokine analysis of antigen-specific T cells, all cells expressing CD69 plus one or more cytokines were first Boolean gated, and then this overall Ag-responding population was subdivided into the subsets of interest on the basis of surface phenotype or cytokine production pattern^4,19,67^.

### Antibody responses to TR3 in rhesus macaques

End-point titer Enzyme-Linked Immunosorbent Assay (ELISA) was used to quantify anti-HCMV antibodies in plasma from RM. High binding ELISA 96-well plates (Costar) were coated overnight with 100 µl of lysates generated from HCMV TR-infected fibroblasts at 10 µg/ml diluted in PBS. Plates were incubated for 1 hour with 200 µl of blocking buffer (2% milk powder resuspended in PBST (PBS plus 0.1% Tween-20) and then washed three times with PBST. Serial two-fold dilutions of primary plasma samples, starting at 1:100, were generated in blocking buffer and then incubated for 2 h at room temperature. Plates were washed three times with PBST and then incubated with secondary anti-human IgGAM (Rockland) conjugated with horseradish peroxidase. Plates were washed three times with PBST and bound secondary antibody was detected using the OPD substrate (Life Technologies) followed by addition of HCl to stop the reaction. The plates were read at 490 nm using a Synergy HTX Microplate Reader (BioTek). End-point antibody binding titers were calculated by Log/Log transformation and analyzed using GraphPad Prism v6 software.

## Supplementary information

Supplemental information

## Data Availability

The datasets generated during and/or analyzed during the current study are available from the corresponding author on reasonable request.
