# Supplementary material for: Characterization of a live-attenuated HCMV-based vaccine platform
Source: Sci Rep. 2019 Dec 17;9:19236. doi: 10.1038/s41598-019-55508-w (PMC6917771; doi:10.1038/s41598-019-55508-w)
Supplement: Supplementary file 1 — Supplemental information [file 41598_2019_55508_MOESM1_ESM.pdf]

Supplemental information for

Characterization of a live-attenuated HCMV-based vaccine platform

by

Patrizia Caposio[1§], Sjoerd van den Worm[1§2], Lindsey Crawford[1], Wilma Perez[1], Craig Kreklywich[1], Roxanne M. Gilbride[1], Colette M. Hughes[1], Abigail B. Ventura[1], Robert Ratts[1,3], Emily E. Marshall[1,3], Daniel Malouli[1], Michael K. Axthelm[1], Daniel Streblow[1], Jay A. Nelson[1], Louis J. Picker[1], Scott G. Hansen[1*] and Klaus Früh[1*]

[1]Vaccine and Gene Therapy Institute, Oregon Health & Science University, Beaverton, Oregon, 97006, USA.

[2]Current Address: Batavia Biosciences B.V., Zernikedreef 16, 2333 CL Leiden, Netherlands

[3]Current Address: Vir Biotechnology, 4640 SW Macadam Avenue Portland, OR 97239

*Corresponding authors:

Klaus Früh, PhD, Scott G. Hansen, PhD

Vaccine & Gene Therapy Institute

Oregon Health & Sciences University

505 NW 185th Ave

Beaverton, OR, 97006

phone: 503 418 2700

email: fruehk@ohsu.edu, hansensc@ohsu.edu

§These authors contributed equally to this work.

Supplemental Figures

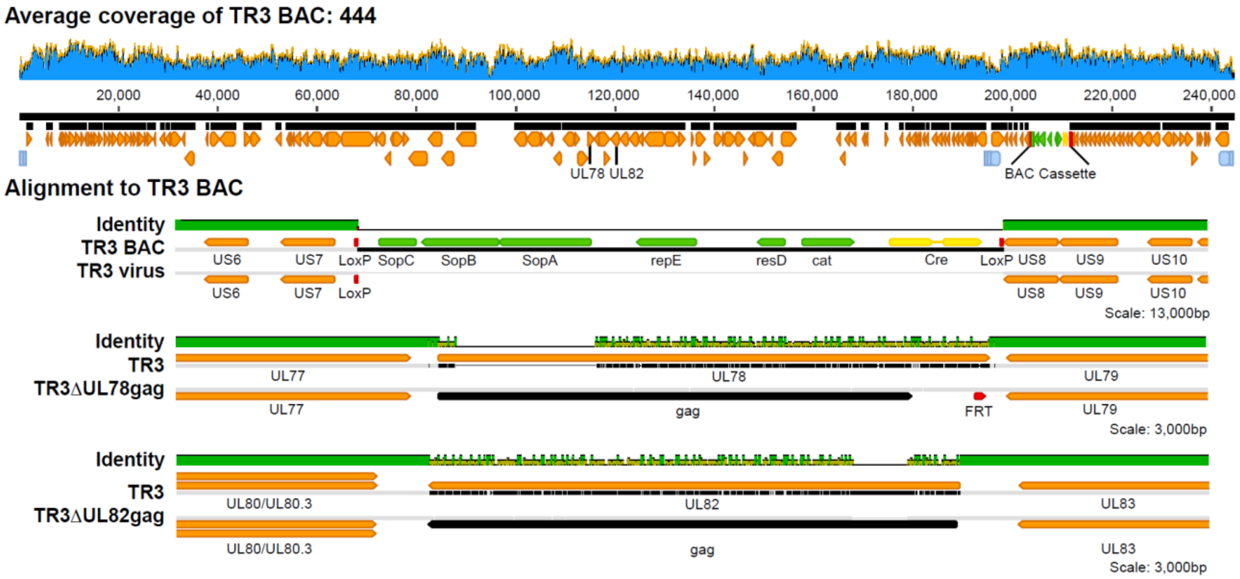

Figure S1: Sequence analysis of TR3 (BAC and Virus), TR3ΔUL78gag and TR3ΔUL82gag. The top panel shows the sequencing coverage map for the TR3 (BAC) graphically depicted as number of reads per nucleotide position. The ORF map is shown below with the BAC cassette highlighted in green and cre-gene in yellow. Genome alignments of TR3 (BAC) with TR3-virus as well as TR3 (BAC) with TR3ΔUL78gag (BAC) and TR3ΔUL82gag (BAC) are shown below. Only the regions that differ between TR3 (BAC) and the indicated recombinants are shown. The green bar indicates the percentage of nucleotide identity between both virus sequences with green being 100% identical. The black bar represents the HIVgag ORF. The residual FRT site in TR3ΔUL78gag is shown in red.

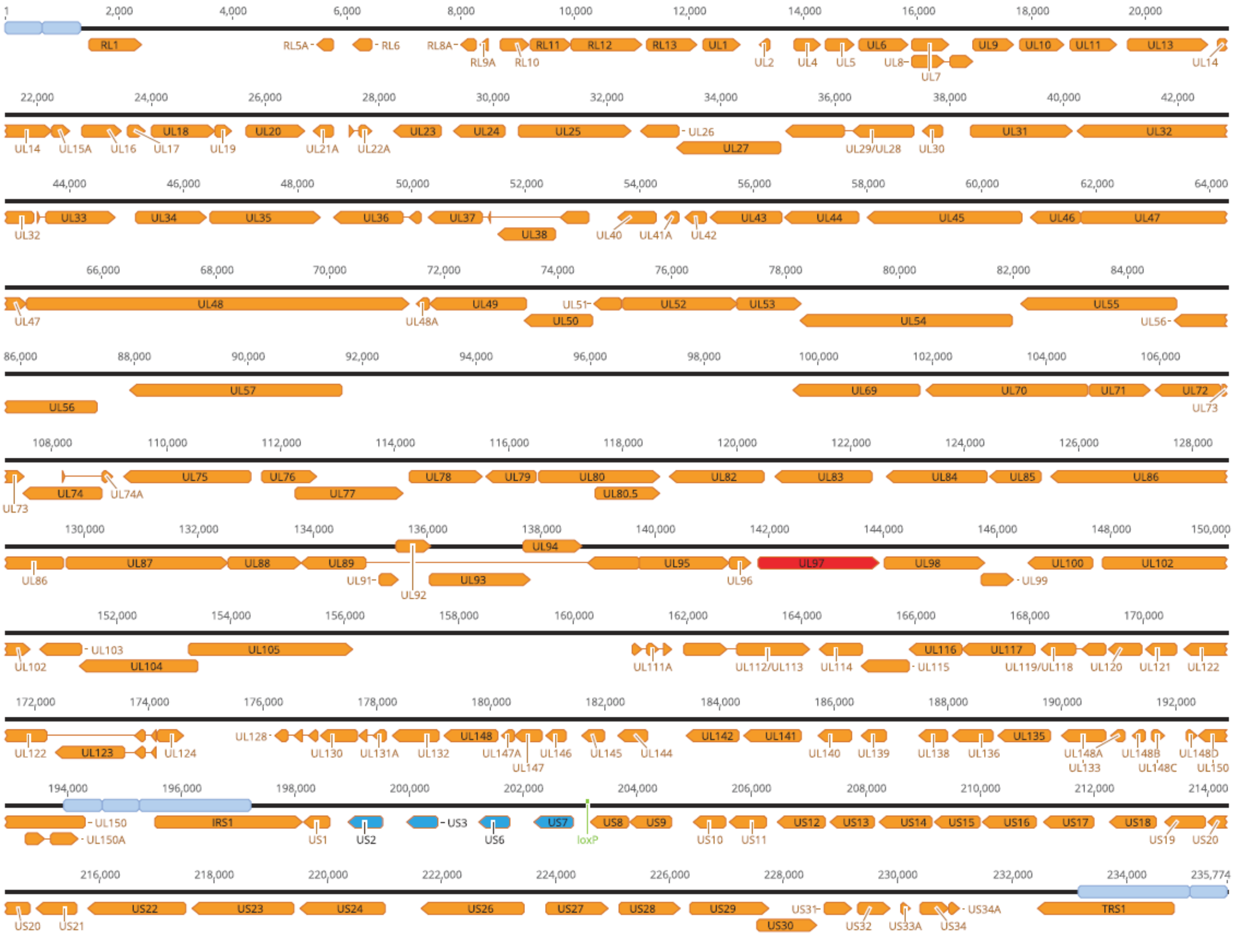

Figure S2: ORF map of HCMV TR3 (BAC). Orange: viral ORFs, light blue: repeat region, red: UL97 from HCMV AD169, dark blue: US2-US7 from HCMV AD169.

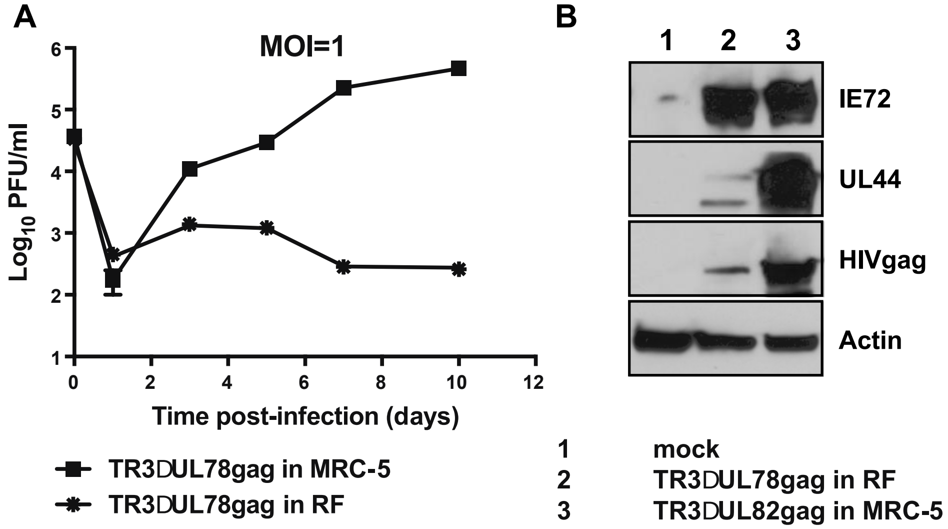

Figure S3: HCMV TR3 does not replicate in rhesus fibroblasts. A) Growth curve of TR3 in human versus rhesus fibroblasts. MRC-5 cells or primary rhesus fibroblasts were infected with TR3ΔUL78gag at an MOI=1. Supernatants were collected at the indicated days and the viral titer determined. B) Viral protein expression in human versus rhesus fibroblasts. MRC-5 cells or primary rhesus fibroblasts were infected with TR3ΔUL78gag (MOI=1) and infected cells were harvested at 96h post-infection. Cell lysates were electrophoretically separated and probed for the indicated proteins by immunoblot.

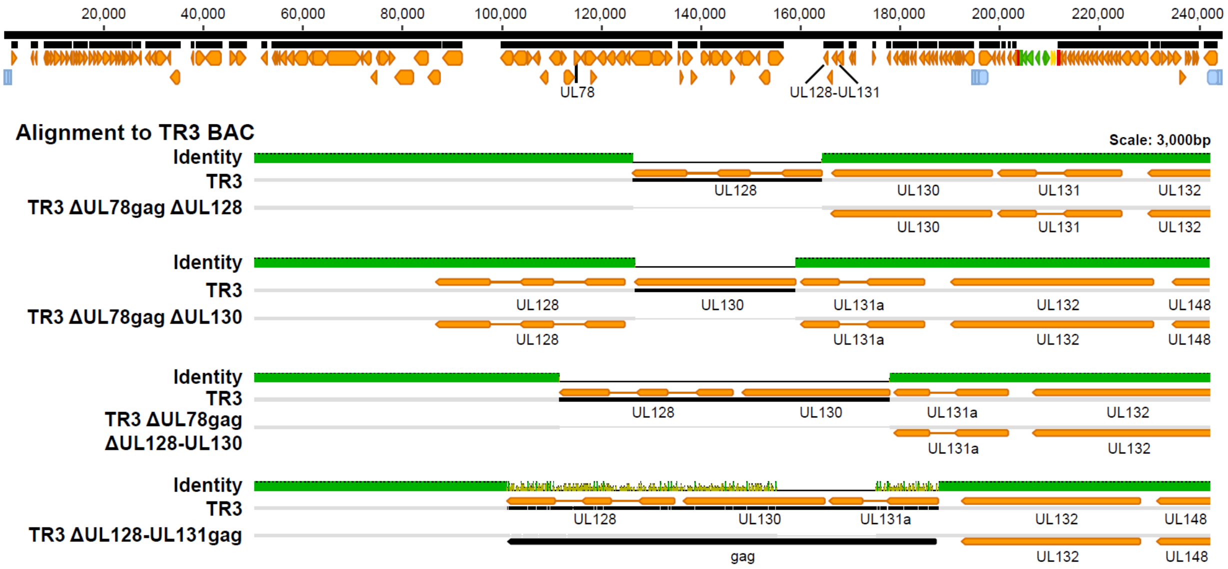

Figure S4: Sequence analysis of PC-deficient HCMV TR3 vectors. The top panel shows the ORF map of TR3 (BAC) with the BAC cassette and modified ORFs highlighted. Genome alignments of TR3 (BAC) with the indicated recombinants are shown below. Only the region spanning the UL128-131 ORFs is shown. The green bar indicates the percentage of nucleotide identity between both virus sequences with green being 100% identical. TR3ΔUL78gag was used to delete UL128 and/or UL130 to generate TR3ΔUL78gagΔUL128, TR3ΔUL78gagΔUL130 and TR3ΔUL78gagΔUL128-130, whereas HIVgag replaces the UL128-131 region in TR3ΔUL128-131gag as indicated.

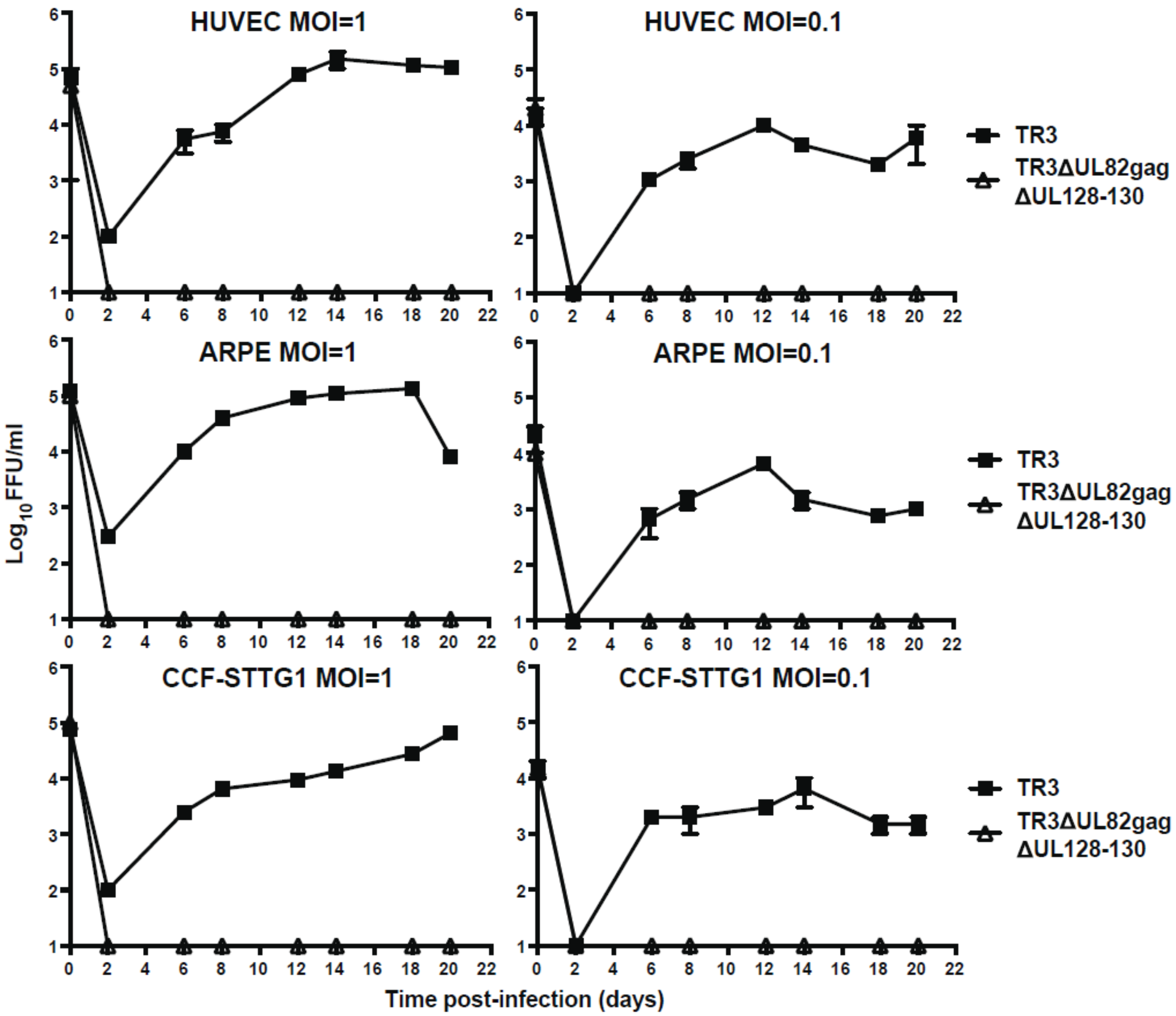

Figure S5: Pentamer-and pp71-deficient TR3 does not grow in non-fibroblast cells. Endothelial cells (HUVEC), epithelial cells (ARPE-19) and astrocytic cells (CCF-STGG1) were infected with the indicated MOI of TR3 or TR3ΔUL82gag. The supernatants were harvested at the indicated days and the virus titer determined on BJ-pp71 cells.

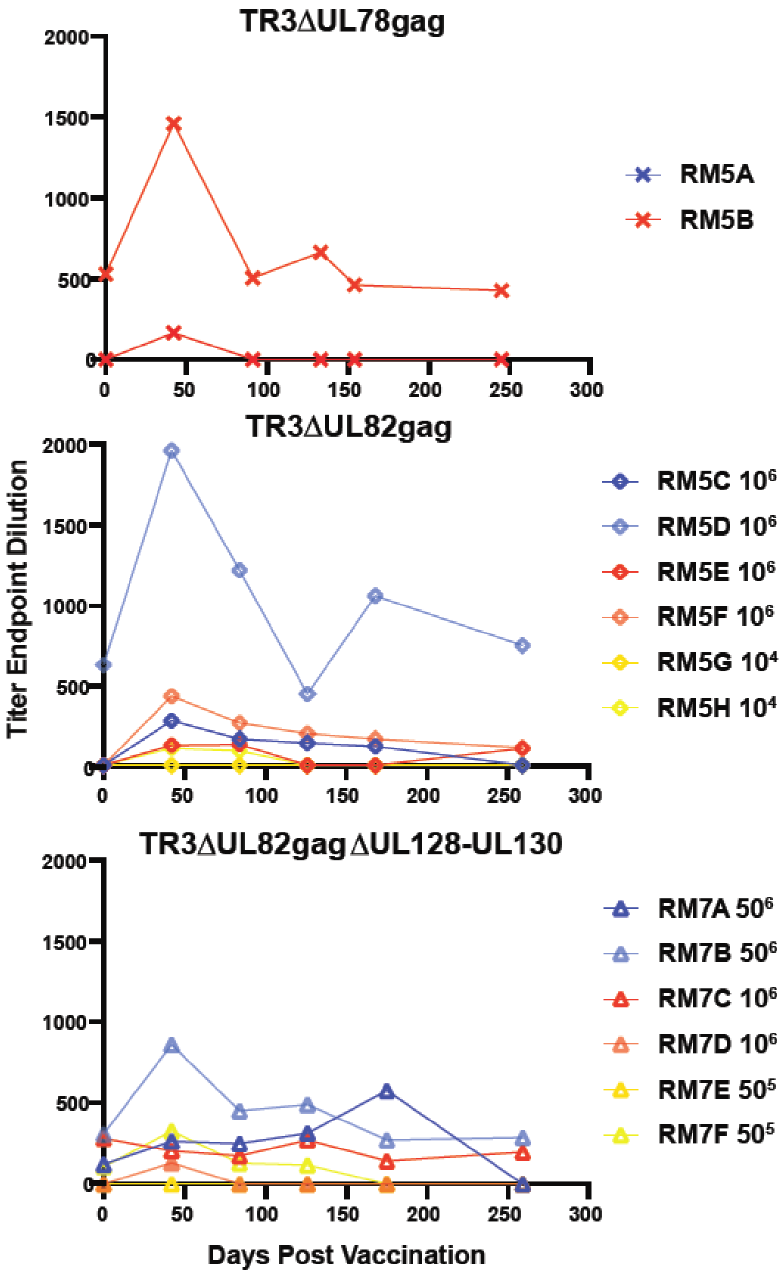

Figure S6: Anti-HCMV antibody responses elicited by PC-intact and PC-deficient UL82-deleted TR3. As shown in Figures 5 and 7, RM were inoculated with TR3ΔUL78gag, TR3ΔUL82gag or TR3ΔUL82gagΔUL128-130 at the indicated doses. Shown are end-point titers for the indicated days post-inoculation and determined by ELISA using HCMV-infected cell lysate.

Supplemental Data: Immunoblots used in this study

Figure 1C

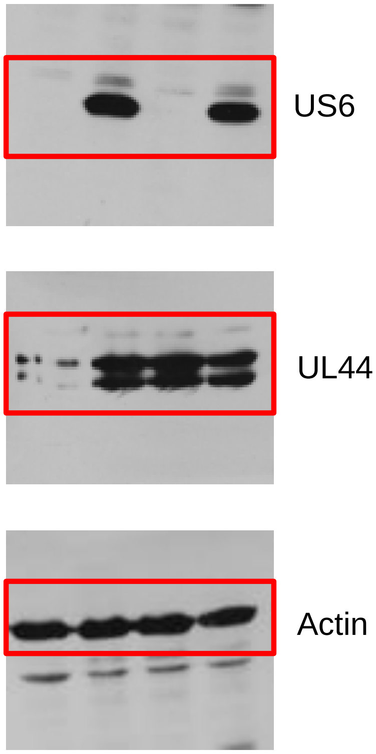

Figure 3A

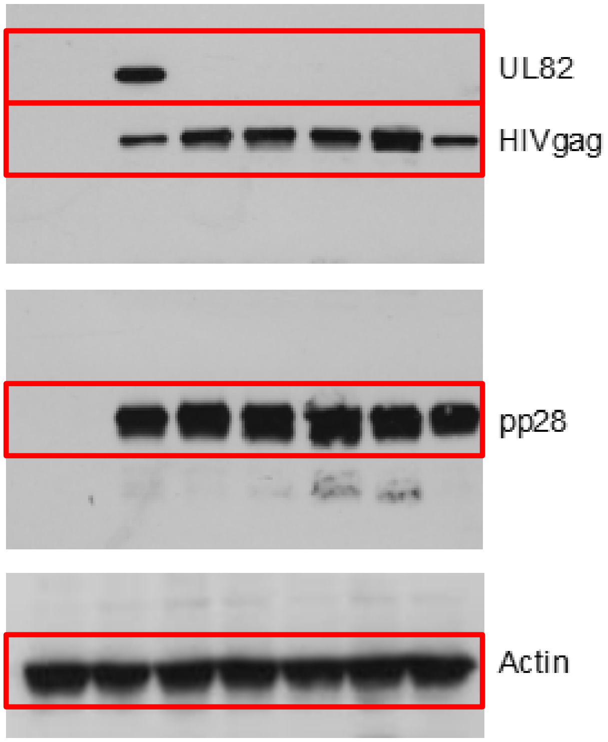

Figure IE

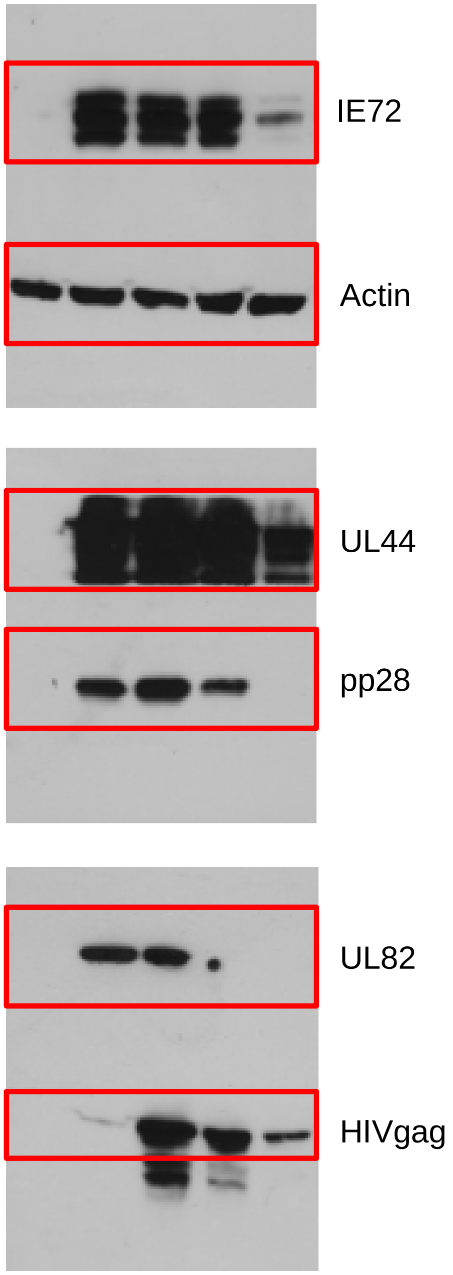

Figure 7A

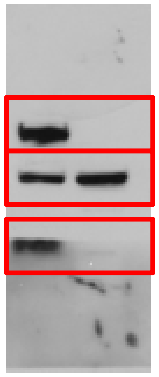

UL82

HIVgag

UL130

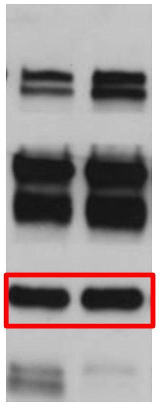

IE72 (data not shown in the manuscript)

UL44 (data not shown in the manuscript)

pp28

Figure S3

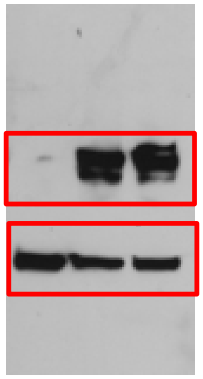

IE72

Actin

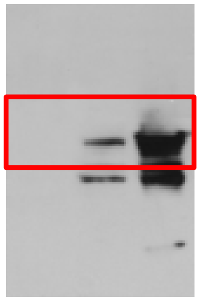

HIVgag

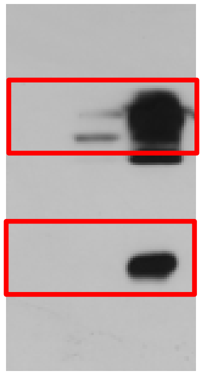

UL44

pp28 (data not shown in the manuscript)
